# GAPDH controls extracellular vesicle biogenesis and enhances the therapeutic potential of EV mediated siRNA delivery to the brain

**DOI:** 10.1038/s41467-021-27056-3

**Published:** 2021-11-18

**Authors:** Ghulam Hassan Dar, Cláudia C. Mendes, Wei-Li Kuan, Alfina A. Speciale, Mariana Conceição, André Görgens, Inna Uliyakina, Miguel J. Lobo, Wooi F. Lim, Samir EL Andaloussi, Imre Mäger, Thomas C. Roberts, Roger A. Barker, Deborah C. I. Goberdhan, Clive Wilson, Matthew J. A. Wood

**Affiliations:** 1 Department of Paediatrics, University of Oxford, Oxford, OX1 3QX UK; 2 Department of Physiology, Anatomy and Genetics, University of Oxford, Oxford, OX1 3QX UK; 3 John van Geest Centre for Brain Repair, Department of Clinical Neurosciences, University of Cambridge, Cambridge, CB2 0PY UK; 4 Department of Laboratory Medicine, Clinical Research Center, Karolinska Institutet, 14186 Stockholme, Sweden; 5 MDUK Oxford Neuromuscular Centre, University of Oxford, Oxford, OX2 9DU UK; 6 Oxford-Harrington Rare Disease Centre, University of Oxford, Oxford, OX2 9DU UK

**Keywords:** RNAi therapy, Extracellular signalling molecules

## Abstract

Extracellular vesicles (EVs) are biological nanoparticles with important roles in intercellular communication, and potential as drug delivery vehicles. Here we demonstrate a role for the glycolytic enzyme glyceraldehyde-3-phosphate dehydrogenase (GAPDH) in EV assembly and secretion. We observe high levels of GAPDH binding to the outer surface of EVs via a phosphatidylserine binding motif (G58), which promotes extensive EV clustering. Further studies in a *Drosophila* EV biogenesis model reveal that GAPDH is required for the normal generation of intraluminal vesicles in endosomal compartments, and promotes vesicle clustering. Fusion of the GAPDH-derived G58 peptide to dsRNA-binding motifs enables highly efficient loading of small interfering RNA (siRNA) onto the EV surface. Such vesicles efficiently deliver siRNA to multiple anatomical regions of the brain in a Huntington’s disease mouse model after systemic injection, resulting in silencing of the huntingtin gene in different regions of the brain.

## Abstract (web summary)

GAPDH is generally considered a housekeeping gene and functions in glycolysis. Here, the authors show that GAPDH has a role in promoting vesicle clustering in endosomes and can load siRNA onto the surface of extracellular vesicles, which can be exploited therapeutically.

## Introduction

Extracellular vesicles (EVs) are emerging as important mediators of intercellular communication by which cells can exchange information in the form of lipids, proteins or nucleic acids^1^. EVs are lipid bilayer-enclosed nanoscale particles that contain membrane-associated proteins and cytosolic components derived from the parent cell^2^. There are several classes of EVs, but small EVs with a diameter of 30–150 nm are the most well-studied^3^. They are secreted by cells as exosomes formed in intracellular endosomal compartments or as microvesicles shed from the cell surface. Given their unique biological and pharmacological characteristics, EVs have attracted interest as mediators of physiological and pathophysiological processes, and have potential as cell-free therapeutics^4^. EVs have been reported to play important roles in the regulation of the immune response^5^, metastasis of cancer cells^6,7^, propagation of pathogenic proteins involved in neurodegenerative disorders^8^, and are emerging as a source of biomarkers for a variety of diseases^9^.

Although heterogeneity of EVs has complicated their molecular characterization, significant advances have been made in our understanding of their biogenesis in recent years^10^. Multiple mechanisms of exosome and microvesicle biogenesis have been identified that involve endosomal sorting complexes required for transport (ESCRT)-dependent and ESCRT-independent processes. Multiple cellular components have been identified that participate in these processes including Alix, tumour susceptibility gene 101 protein (TSG101), syndecan-syntenin complexes, the tetraspanin family and lipid rafts^11,12^. However, we still lack a detailed understanding of the regulatory and loading processes involved.

EVs have also attracted considerable interest as potential vehicles for drug delivery, particularly as carriers of macromolecules like non-coding RNAs and proteins^13^. Their immunological inertness and ability to cross biological barriers are two important features that can be exploited for therapeutic applications^14^. EVs have been demonstrated to deliver therapeutic small interfering RNAs (siRNAs) and antisense oligonucleotides in multiple therapeutic contexts, including mouse models resulting in suppression of pancreatic cancer and restoration of dystrophin expression for the treatment of muscular dystrophy, respectively^15–17^. However, an incomplete understanding of EV biogenesis and uptake mechanisms, and a lack of efficient drug loading methods remain critical challenges that need to be addressed^18,19^. Current methods of loading therapeutic molecules into EVs such as electroporation, genetic engineering of host cells and chemical conjugation, are limited by low efficiency, toxicity and lack of scalability^20,21^. Moreover, they produce a heterogeneous population of EVs that generates further complexity in terms of understanding the phenotypic effects of EVs in target cells^22^. Development of methodologies in which the natural properties of EVs are exploited for therapeutic applications is likely to provide a more robust approach to this problem.

During the last two decades, studies of glyceraldehyde-3-phosphate dehydrogenase (GAPDH), traditionally known as a ‘housekeeping’ protein, have revealed multiple functions in cellular processes that are independent of its canonical role in glycolysis. Earlier studies demonstrated that GAPDH is a nucleic acid binding protein that prevents rapid shortening of telomeric DNA, is involved in transport of tRNA and mRNA, regulates expression of genes via specific binding to the 5′UTR and 3′UTR of mRNA and possesses uracil DNA glycosylase activity involved in repairing damaged DNA^23–25^. GAPDH is also a critical mediator of cellular responses to oxidative stress. Under low oxidative stress, GOSPEL (GAPDH’s competitor of Siah protein enhances life) preserves glycolytic function of GAPDH and prevents its S-nitrosylation. High oxidative stress levels induce S-nitrosylation of GAPDH, leading to its interaction with Siah and subsequent nuclear translocation, resulting in ubiquitylation and degradation of nuclear proteins, triggering apoptosis^26,27^.

Recent reports have revealed complex and paradoxical roles for GAPDH in preventing caspase-independent cell death (CICD) in cancer cells. Overexpression of GAPDH stabilizes active Akt, which in turn leads to overexpression of anti-apoptotic Bcl-xL, which protects mitochondria permeabilization^28^. GAPDH also participates in upregulation of autophagy protein ATG12, which enhances autophagy and enables clearance of damaged mitochondria, preventing CICD, which in turn leads to clonogenic outgrowth of cells^29^. Besides, its diverse physiological roles, GAPDH is also involved in several neurological diseases. GAPDH has been identified as a major component of amyloid plaques in Alzheimer’s disease and is known to interact with amyloid −β protein precursors^30^. The expanded polyglutamine repeats of mutant huntingtin protein have been shown to associate with GAPDH, potentially disrupting GAPDH-mediated trafficking of damaged mitochondria into the lysosomal system^31^.

The functional diversity of GAPDH is attributed to its oligomeric nature, post-translational modifications and compartmentalization, allowing it to interact with different proteins^32^. GAPDH is one of the most abundant proteins associated with EVs, and has been reported to bind to the EV outer membrane, where it interacts with the iron-binding proteins transferrin and lactoferrin and traffics them into cells from tissue culture medium^33^. Recently, it has been observed that GAPDH becomes associated with exosomes during formation of intraluminal vesicles and that exocytosis of exosomes represents one pathway for secretion of GAPDH from cells, a process that is enhanced when cells are depleted of iron^34^.

Here, we show that GAPDH associates with EVs via a phosphatidylserine-binding motif, and that the addition of exogenous GAPDH to EV preparations induces EV clustering. Analysis in an in vivo *Drosophila* model of exosome biogenesis in the recycling endosomal compartments reveals that endogenous GAPDH is involved in exosome clustering and the formation of central dense granules in multi-vesicular endosomes, and also affects exosome formation and secretion. Furthermore, chimeric EV-binding G58 proteins that lack clustering activity, but are fused with an RNA-binding domain, were shown to facilitate the siRNA loading onto the EV surface and exhibit enhanced gene silencing activity in cell culture, and in a mouse model of Huntington’s disease.

## Results

### GAPDH binds cleaved lactoferrin N at the EV surface

**Fig. 1:**
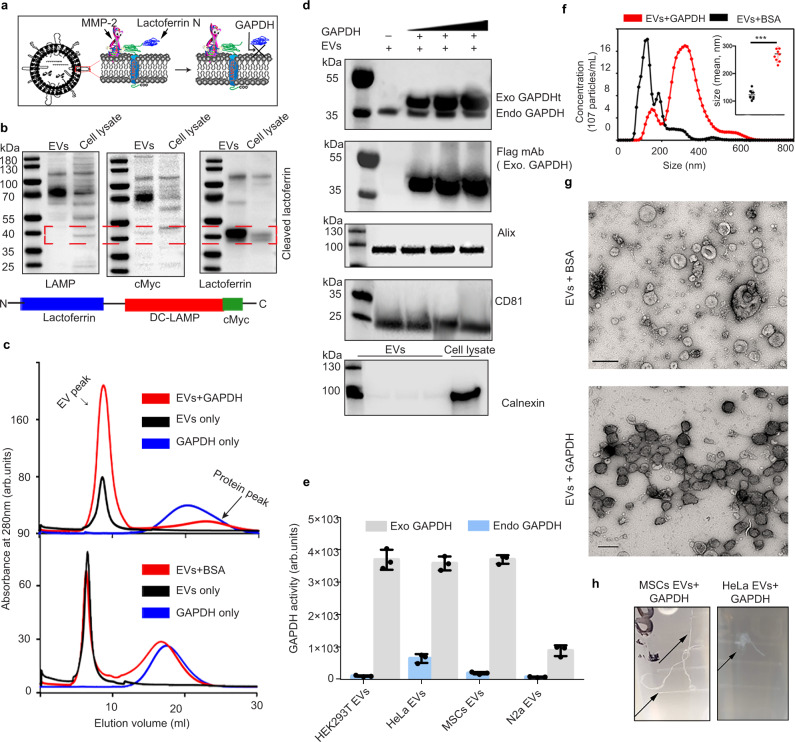
Surface binding of GAPDH leads to aggregation of EVs. **a** Cartoon representation of lactoferrin N cleavage from DC-LAMP by MMP-2 enzyme, and attachment of the cleaved lactoferrin on the EV surface via GAPDH protein. **b** Western blotting of purified HEK293T EVs and cell lysate showing expression of lactoferrin N-DC-LAMP and cleavage of lactoferrin from DC-LAMP (Dendritic cell-Lysosomal associated membrane protein). Arrangement of the different domains of the protein is shown in the schematic. The experiment was independently repeated two times. **c** UV-absorbance spectrum for EVs, EVs+GAPDH and EVs+BSA after passing through gel-filtration column. GAPDH and BSA protein alone were used as negative controls to rule out aggregation of the proteins. The experiment has been independently repeated three times. **d** Western blot showing exogenous binding of GAPDH to HEK293T EVs. Increasing concentrations of histidine-(His6) and Flag-tagged GAPDH were incubated with a fixed number of EVs. Endo GAPDH Endogenous GAPDH, Exo GAPDH exogenous GAPDH. Alix and CD81 are EV protein markers used as positive controls. Calnexin (bottom blot) was used to demonstrate the purity of EV samples. The experiment has been independently repeated two times. **e** GAPDH activity assay of EVs and EVs+Exo GAPDH. For EVs+Exo GAPDH activity, GAPDH protein was added to EVs in concentrated (10×) complete tissue culture media (Media + 10% FBS) followed by gel filtration to isolate EVs. Results are shown as mean ± s.d (*n* = 3 independent biological experiments). **f** NTA profile showing the size distribution of purified HEK293T EVs after incubation with either GAPDH or BSA proteins respectively. Inset is the scatter plot representing size (mean) of EVs. Data shown as mean ± s.d. (*n* = 3 independent biological experiments) Statistical differences were determined by unpaired two-sided Student’s t-test. ***P < 0.0001. **g** Electron microscopy images of EVs incubated with either BSA or GAPDH protein (scale bar indicates 200 nm). **h** Photographic images of tubes showing the formation of thread-like structures from MSCs and HeLa EVs after incubation with GAPDH for 2 h at 4 °C. Experiment of (**g** and **h**) were independently repeated two times. Source data are provided as a Source Data file.

In a set of experiments designed to express the N-terminal region of lactoferrin (hereafter lactoferrin N) on the surface of EVs for the purposes of brain targeting^35^, we observed that lactoferrin N, which was attached to the EV surface via fusion to DC-LAMP (Dendritic cell lysosomal associated membrane glycoprotein), was specifically cleaved from this anchor. Surprisingly, cleaved lactoferrin N (which lacks signal peptide and membrane-associated domain) remained associated with the EV surface (Fig. 1a, b). Previously, it was demonstrated that cells take-up extracellular iron by secreting EVs carrying surface GAPDH, which interacts with the iron-binding proteins lactoferrin and transferrin^33^. Informed by these findings, we sought to determine whether GAPDH is involved in anchoring lactoferrin to the EV membrane. Using a protease digestion assay, we observed the presence of GAPDH on the outer surface of EVs isolated from HEK293T, HeLa, MSCs, SKOV-3 and B16 lymphoma cells (Supplementary Fig. 1a & b). EV-associated GAPDH was found to be enzymatically active (Supplementary Fig. 1c). Co-immunoprecipitation experiments of HEK293T cell lysates and EVs demonstrated that the lactoferrin N domain is physically associated with GAPDH in cells and on the surface of EVs (Supplementary Fig. 1d). Furthermore, incubation of isolated EVs with purified lactoferrin N protein resulted in efficient binding of the protein to the EV surface. However, incubating purified EVs with a different domain of lactoferrin, the N1.1 domain, which lacks the GAPDH binding motif, did not result in binding to the EV surface (Supplementary Fig. 1e & 2a). Taken together, these experiments demonstrate the normal role of GAPDH in tethering lactoferrin N on the surface of EVs.

### Binding of GAPDH to EVs causes EV clustering

We next investigated whether additional GAPDH molecules could be loaded onto EVs. Although overexpression of GAPDH in secreting cells had a minimal effect on GAPDH loading, incubation of isolated EVs with purified GAPDH resulted in extensive surface binding of GAPDH, as confirmed by immunoblotting, gel filtration and GAPDH enzymatic activity (Fig. 1c–e). The lack of an ‘EV’ peak after loading GAPDH protein alone confirmed the absence of any GAPDH aggregates in the sample (Fig. 1c). Binding of GAPDH to EVs occurred for all cell sources tested, and the presence of serum proteins in the tissue culture media did not affect binding of GAPDH to EVs (Fig. 1e & Supplementary Fig. 2b). Interestingly, incubation of EVs with GAPDH resulted in an increase in EV particle size, as determined by nanoparticle tracking analysis (Fig. 1f). Electron microscopy of HEK293T EVs after incubation with GAPDH revealed the formation of long branched chains of EVs (Fig. 1g). EVs derived from HeLa, B16-F10 and mesenchymal stromal cells (MSCs) formed pronounced thread-like structures when incubated with GAPDH protein, suggesting that GAPDH induces EV clustering (Fig. 1h & Supplementary Fig. 2d,e). An electron dense corona of GAPDH was observed, tethering the EVs (Supplementary Fig. 2d). The width of this tether was found to be around 13.54 ± 5.5 nm (mean ± s.d, *n* = 30). Interestingly, it has recently been observed that newly formed intraluminal vesicles (ILVs) from the limiting membrane of some multi-vesicular bodies (MVBs), the compartments that produce exosomes, are physically attached to each other via electron dense tethers with an approximate width of ~12 nm^36^. However, the biochemical nature of this tether remains to be determined, although it is tempting to speculate that GAPDH is involved.

### GAPDH binds to EVs via a phosphatidylserine-binding G58 domain

**Fig. 2:**
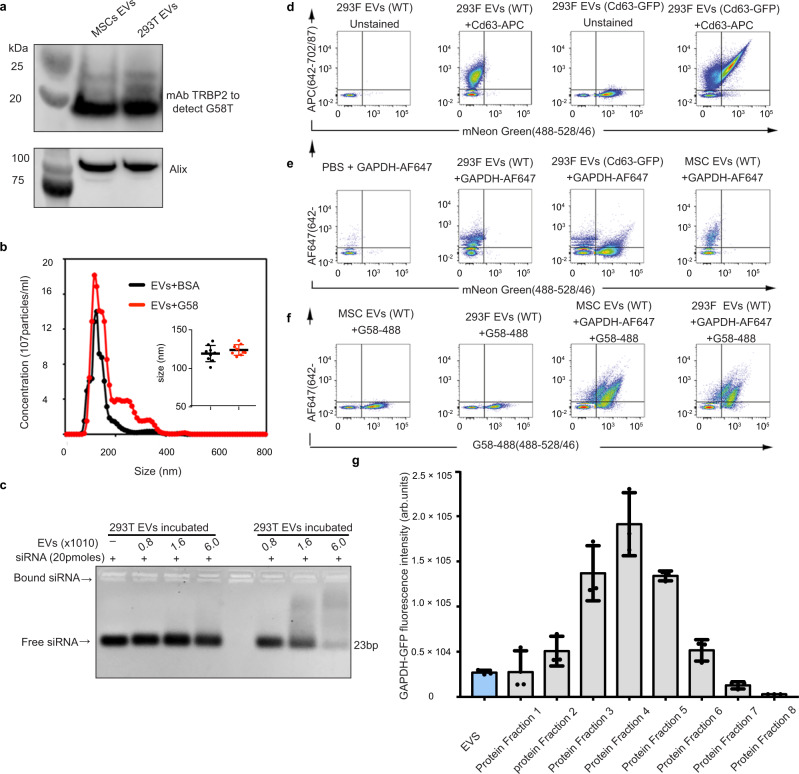
GAPDH binds to EV surface via G58 domain. **a** Western blot showing binding of G58 peptide to HEK293T (designated as 293T) and MSC EVs. The second domain of TARBP protein was attached to G58 peptide for detection by anti-TARBP2 antibody. The experiment was independently repeated two times. **b** NTA profile showing the size distribution of HEK293T EVs after binding to the G58T protein. Inset is the scatter plot representing size (mean) of EVs. Each dot is a mean of three reading frames taken at different timepoints. Data are shown as mean ± s.d (*n* = 9 independent biological experiment) Statistical differences were determined by unpaired two-sided Student’s *t*-test, (ns = non-significant). **c** Agarose-gel-shift assay of EVs after incubation with either G58T (G58 + dsRBD) protein or dsRBD of TARBP2 protein. siRNA alone was used as negative control to determine interaction of EVs with siRNA. A gradual decrease in the intensity of siRNA reflects entrapment of siRNA near the wells due to interaction with G58T EVs. Lack of siRNA binding to dsRBD treated EVs confirms G58 peptide mediated binding of protein to EV surface. The experiment was independently repeated three times. **d**–**f** High-resolution single EV analysis by Imaging Flow Cytometry (IFC) to determine localization of GAPDH and G58 peptide on EVs. **d** Represents method validation by using either non-labelled HEK293F derived EVs or neon GFP labelled HEK293:CD63-neon GFP derived EVs as biological reference material. **e** Detection of GAPDH on HEK293F, HEK293F/CD63-GFP and MSCs EVs, using alexa fluor 647 labelled anti-GAPDH antibody. **f** G58 peptide binding on EVs expressing GAPDH on their surface. EVs were incubated with alexa fluor 488 (af488) labelled G58 peptide and af647 anti-GAPDH antibody. Experiment (**d**–**f**) were independently repeated three times. FACS sequential gating/sorting strategies is provided as Supplementary Fig. 9. **g** Distribution of secreted GAPDH-GFP protein in the cell-culture media. Media from HEK293T cells expressing GAPDH-GFP protein were processed to isolate EVs from proteins by gel-filtration chromatography. Both EVs and protein fractions contained GAPDH-GFP protein, indicating vesicular and non-vesicular modes of GAPDH secretion. Data are shown as mean ± s.d (*n* = 3 independent biological experiments). Source data are provided as a Source Data file.

GAPDH isolated from rabbit brain tissues has been shown to induce fusion of synthetic lipid vesicles that contained phosphatidylserine (PS), cholesterol and plasmenylethanolamine^37^. Interestingly, the fusogenic activity of GAPDH has also been reported to play an important role in nuclear membrane formation^38^. GAPDH protein has been reported to bind to the nuclear membrane via a conserved PS-binding domain, located between amino acids 70–94 (Supplementary Fig. 2c)^39^. Since PS is present on the outer surface of EVs^40^, we next investigated whether the G58 peptide is also responsible for binding of GAPDH to EVs. To this end, we generated a fusion construct consisting of the PS-binding motif of GAPDH (designated as G58) and the double-stranded RNA-binding domain (dsRBD) of TARBP2 (TAR RNA-binding protein 2), which could be detected with an anti-dsRBD antibody. This chimeric protein (designated as G58T) was expressed in, and purified from, *E.coli* cells. Purified G58T protein was incubated with EVs for 2 h at 4 °C, and unbound protein was separated from EVs by gel-filtration chromatography. Western blotting and gel-binding assays revealed extensive binding of G58 peptide to the EV surface (Fig. 2a). However, binding of G58 did not significantly alter the size of EVs (Fig. 2b), suggesting that sequences outside of the GAPDH G58 motif, which are presumably required for tetramerization, are involved in EV clustering. Moreover, Incubation of EVs with recombinant dsRBD of TARBP2 did not result in EV binding, suggestive of specific binding of G58 peptide to the EV surface (Fig. 2c) Quantification of G58 binding on MSC- and HEK293T-derived EVs revealed ~1200 and 1400 G58 peptide binding sites per EV, respectively (Supplementary Fig. 2f, g).

To determine whether other domains of GAPDH are involved in mediating binding and clustering of EVs, we generated different GAPDH mutants (Supplementary Fig. 3a). In the G150T mutant, the NAD^+^ binding domain (1–150 amino acid) of GAPDH that contains the PS-binding motif was fused to second domain of TRBP2 protein and the recombinant DNA clone was expressed in *E. coli* cells (Supplementary Fig. 3b). Incubation of purified G150T protein with EVs resulted in efficient binding of the protein to the surfaces of EVs, confirming that the catalytic domain of GAPDH protein is not required for binding to EVs (Supplementary Fig. 3c, d). In the G∆(71–90) mutant, we deleted the PS-binding domain of GAPDH but for unknown reasons we could not express the protein in cells. To further characterize binding of GAPDH to the EV surface, we mutated two important amino acid residues of GAPDH (R80/A80 and K84/A84) within the PS-binding domain that have been suggested earlier to have a possible role in binding to PS lipids^41^. Substitution of R80 and K84 to alanine did not affect expression levels of the mtGAPDH protein produced, and it had similar glycolytic activity to wtGAPDH (Supplementary Fig. 3e–g). However, we found a significant decrease in EV binding of mtGAPDH when compared to wtGAPDH (Supplementary Fig. 3h–k). Altogether, these results confirm the presence of a specific PS-binding domain within the NAD^+^ binding domain. Importantly, the ability of specific amino acid changes to alter binding of GAPDH to the EV surface without affecting glycolytic activity of the enzyme suggests that the PS-binding domain is not required to mediate binding to NADH or glyceraldehyde-3-phosphate, or for the catalytic activity of the enzyme.

To assess whether GAPDH and G58 proteins bind to EVs expressing canonical markers such as CD63, we next analyzed EVs derived from HEK293F cells and MSCs by single vesicle high-resolution Imaging Flow Cytometry (IFC). This method was previously optimized extensively for detection and quantification of single fluorescently labelled EVs with an Amnis Image StreamX MkII instrument^42^. By staining CD63-neon GFP-tagged EVs with APC-labelled anti-CD63 antibodies, we confirmed that the Amnis Cellstream facilitates detection of single fluorescent EVs (Fig. 2d). Endogenous GAPDH was detected on both HEK293F cell and MSC-derived EVs (Fig. 2e). Incubation of EVs from both cell lines with anti-GAPDH antibodies and fluorescently labelled G58 peptide resulted in detection of co-labelled EVs **(**Fig. 2f), thereby confirming on the single EV level that GAPDH is present on EVs derived from both cell lines and that G58 binds extensively to those EVs.

To determine whether GAPDH secretion by cells is mediated via EVs or if a non-vesicular route of GAPDH secretion also exists, we expressed a GAPDH-GFP fusion protein in HEK293T cells and isolated EVs from the cell cultured media. Analysis of GAPDH-GFP fluorescence from EVs and non-EV protein fractions reflected predominant association of GAPDH-GFP in the non-EV protein fractions, suggesting the existence of non-vesicular routes of GAPDH secretion, consistent with reports from others (Fig. 2g)^34,43^.

### GAPDH regulates exosome clustering, biogenesis and secretion in vivo

**Fig. 3:**
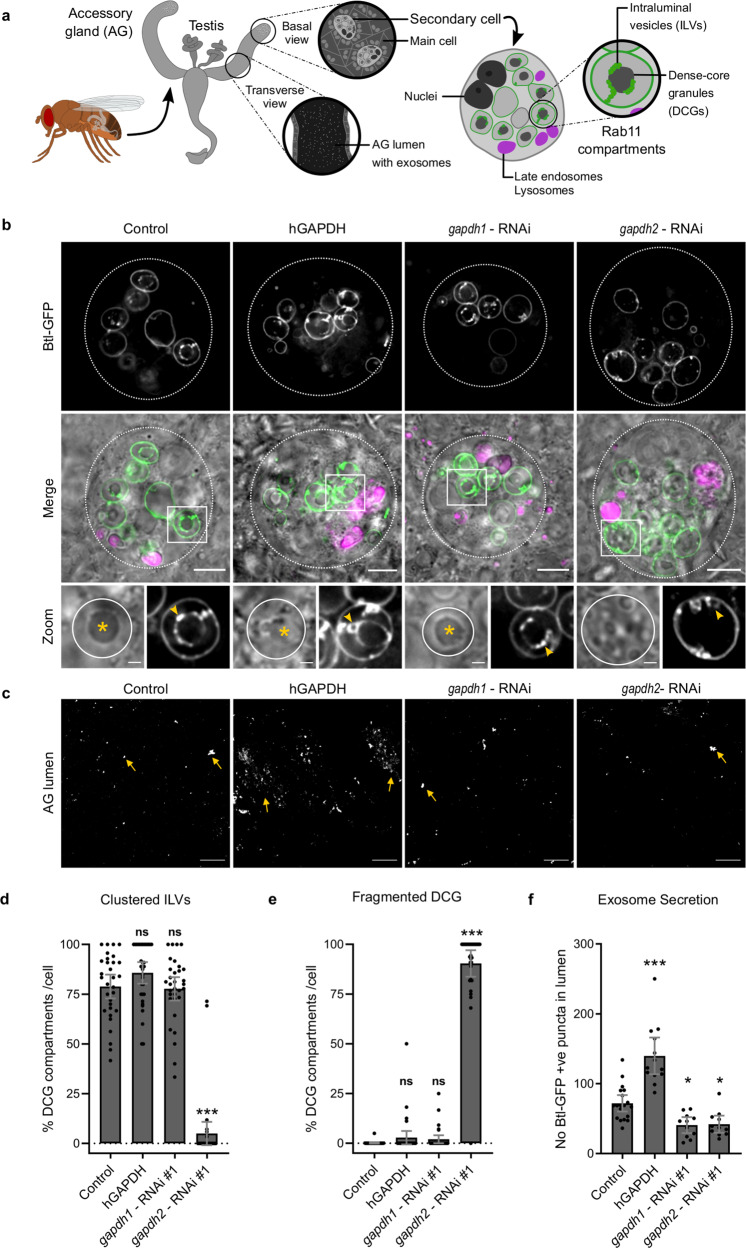
GAPDH regulates exosome biogenesis and clustering in *Drosophila* secondary cells. **a** Schematics show male fruit fly and its accessory gland (AG) containing main cells and secondary cells (SCs), which are only found at the distal tip of the gland. Exosomes can be visualized at the AG lumen as fluorescent puncta. A schematic of a secondary cell expressing a GFP-tagged form of Breathless (Btl-GFP; green) is also shown. The Rab11 compartments, which contain intraluminal vesicles (ILVs; green) and dense-core granules (DCGs; dark grey), and the late-endosomes and lysosomes (magenta) are marked. **b** Basal wide-field fluorescence and differential interference contrast (‘Merge’) views of living secondary cells (SCs) expressing GFP-tagged form of Breathless (Btl-GFP; green) with no other transgene (control); or also expressing the open reading frame of the human GAPDH protein (hGAPDH), an RNAi construct targeting *Drosophila* GAPDH1 (*gapdh1*–RNAi #1), or an RNAi construct targeting *Drosophila* GAPDH2 (*gapdh2*–RNAi #1). SC outline approximated by dashed white circles, and acidic compartments are marked by LysoTracker Red (magenta). Btl-GFP-positive intraluminal vesicles (ILVs; green in ‘Merge’; grey in ‘Zoom’) are apparent inside compartments, surrounding dense-core granules (DCGs; asterisk in ‘Zoom’) and connecting DCGs to the limiting membrane of the compartment (yellow arrowheads, except in *GAPDH2* knockdown, where ILVs only surround peripheral small DCGs). DCG compartment outline is approximated by white circles. **c** Confocal transverse images of fixed accessory gland (AG) lumens from the same genotypes, containing Btl-GFP fluorescent puncta (yellow arrows). **d** Bar chart showing percentage of DCG compartments per cell containing clustered Btl-GFP-positive ILVs that are in contact with DCGs (hGAPDH, *p* = 0.5382; *gapdh1*–RNAi #1, *p* > 0.9999; *gapdh2*–RNAi #1, *p* < 0.0001; Kruskal–Wallis and Dunn correction). **e** Bar chart showing the percentage of DCG compartments per cell containing single spherical DCG (hGAPDH, *p* > 0.9999; *gapdh1*–RNAi #1, *p* = 0.0816; *gapdh2*–RNAi #1, *p* < 0.0001; Kruskal–Wallis and Dunn correction). **f** Bar chart showing number of Btl-GFP fluorescent puncta in the lumen of AGs for the different genotypes (hGAPDH, *p* = 0.0067; *gapdh1*–RNAi #1, *p* = 0.0238; *gapdh2*–RNAi #1, *p* = 0.0298; Kruskal–Wallis and Dunn correction). All data are from six-day-old male flies shifted to 29 °C at eclosion to induce expression of transgenes. Genotypes are: *w; P[w*^*+*^*, tub-GAL80*^*ts*^*]*+; *dsx-GAL4, P[w*^*+*^*, UAS-btl-GFP]/*+ with no expression of other transgenes (control) (*n* = 11 glands/*n* = 31 cells) (*n* = 19 AG lumens for ILV secretion), or with UAS-hGAPDH (*n* = 10 glands/*n* = 33 cells)(*n* = 13 AG lumens), UAS-*gapdh1*-RNAi (*n* = 10 glands*/n* = 32 cells)(*n* = 11 AG lumens) or UAS-*gapdh2*-RNAi overexpression (*n* = 10 glands*/n* = 33 cells)(*n* = 11 AG lumens). Scale bars in (**b**) (5 µm), in ‘Zoom’ (1 µm), and in **c** (20 µm). ***p < 0.001, **p < 0.01, *p < 0.05 and ns non-significant relative to control, Kruskal–Wallis followed by Dunn’s multiple comparison test. Data shown in (**d**–**f**) as mean ± SEM (*n* = 3 independent biological experiments). All experiments shown were independently repeated three times. Source data are provided as a Source Data file.

A previous study has suggested that GAPDH is preferentially loaded on to exosomes when cells are starved of iron^34^. We have recently shown that when human cancer cell lines are subjected to nutrient stress, such as glutamine depletion, they preferentially secrete a subtype of exosomes, called Rab11a-exosomes, from endosomes marked by the small GTPase Rab11a, one of two Rab11 isoforms^44^. Secondary cells (SCs) of the *Drosophila* male accessory gland (AG) form exosomes as intraluminal vesicles (ILVs) in highly enlarged Rab11-positive compartments. These are then secreted into the lumen of the AG, a storage site for seminal fluid^45^. These Rab11-exosomes can be selectively marked by fluorescent transmembrane markers, such as a GFP-tagged form of the FGF receptor, Breathless (Btl-GFP) or the human exosome marker CD63. Interestingly, they form in clusters that surround a large dense-core granule (DCG) of aggregated protein and extend out to the limiting membrane of the Rab11 compartments (Fig. 3a, b). In *Drosophila*, there are two GAPDH isoforms, GAPDH1 and GAPDH2, which are both closely related in sequence to human GAPDH and may have partially redundant functions^46^. Since *Drosophila* GAPDH has similar EV-binding properties to human GAPDH (Supplementary Fig. 4a,b), we tested whether GAPDH might play a role in ILV clustering.

Overexpression of human GAPDH specifically in adult SCs produced larger clusters of Btl-GFP-positive ILVs (Fig. 3b), and increased clustering of Btl-GFP puncta, representing secreted exosomes, in the AG lumen (Fig. 3c). The ILV clustering phenotype was less obvious when SCs were labelled with the CD63-GFP marker, but CD63-GFP puncta were clearly clustered in the AG lumen (Supplementary Fig. 4d). Although the number of ILV-containing compartments was affected by overexpression of human GAPDH (Supplementary Fig. 43 and 4f), the proportion of these compartments that contained ILVs was not significantly altered (Fig. 3d, Supplementary Fig. 4g). We were unable to detect endogenous GAPDH in SCs. However, an antibody recognizing human GAPDH identified this molecule in hGAPDH-overexpressing SCs, in association with membranous structures inside late endosomal and lysosomal SC compartments, suggesting that it traffics into the endolysosomal system in SCs, as it does in human cells (Supplementary Fig. 4i).

To test the role of GAPDH in normal exosome biogenesis, we knocked down the two *Drosophila GAPDH* genes in SCs. There was no significant effect of *GAPDH1* knockdown on ILV DCG biogenesis in these cells, when using either the Btl-GFP or CD63-GFP markers, although exosome secretion was reduced (Fig. 3b, c, f; Supplementary Fig. 4g and 4h). However, *GAPDH2* knockdown led to a severe disruption of DCG formation in SC compartments. There was no central dense core, but multiple small dense-cores formed near the limiting membrane (Fig. 3b, e). Btl-GFP-labelled ILVs were only located in close proximity to the limiting membrane and around the small DCGs (Fig. 3b, d); they did not cluster in non-DCG-associated chains, as seen in normal cells, demonstrating that this process is GAPDH2-dependent. A similar phenotype was observed with CD63-GFP-marked ILVs, where the proportion of compartments making ILVs was also significantly decreased (Supplementary Fig. 3b and 3e). In addition, *GAPDH2* knockdown significantly reduced exosome secretion from these compartments into the AG lumen (Fig. 3f, Supplementary Fig. 4h).

To check that the identity of DCG compartments had not been altered by this manipulation, we knocked down *GAPDH2* in SCs expressing YFP-Rab11 from the endogenous *Rab11* locus. Compartments containing defective DCGs were still labelled with YFP-Rab11 (Supplementary Fig. 5). However, the proportion of these compartments making YFP-Rab11-positive ILVs was significantly decreased and those ILVs formed were closely associated with small DCGs or the limiting membrane of each compartment, confirming that *GAPDH2* knockdown specifically affects ILV clustering and biogenesis. Notably, ILV and DCG phenotypes were observed with two *GAPDH2*–RNAi targeting different sequences, using all three exosome markers, confirming that the phenotypes did not result from an off-target effect (Supplementary Figs. 4g and 5). Importantly, the *GAPDH2* knockdown phenotype was not recapitulated by knocking down other glycolytic enzymes, namely *Phosphoglucomutase 2* (*Pgm2a*), *Phosphoglucose isomerase* (*Pgi*) and *Phosphofructokinase* (*Pfk*), suggesting that the observed effects on exosome biogenesis are not a consequence of general metabolic changes, but rather due to specific reduction in GAPDH2 expression (Supplementary Fig. 6). Overall, our data indicate that the formation and clustering of ILVs and DCG biogenesis in Rab11 compartments of SCs are regulated by GAPDH2 and appear to be functionally linked processes, a result supported by a recent analysis of ESCRT function in SCs^47^. These findings suggest that the EV clustering phenotype observed upon human GAPDH overexpression in SCs and following loading of fly or human GAPDH on human EVs reflects a normal physiological process in some cell types.

### Therapeutic application of free GAPDH binding sites on EVs in drug delivery

**Fig. 4:**
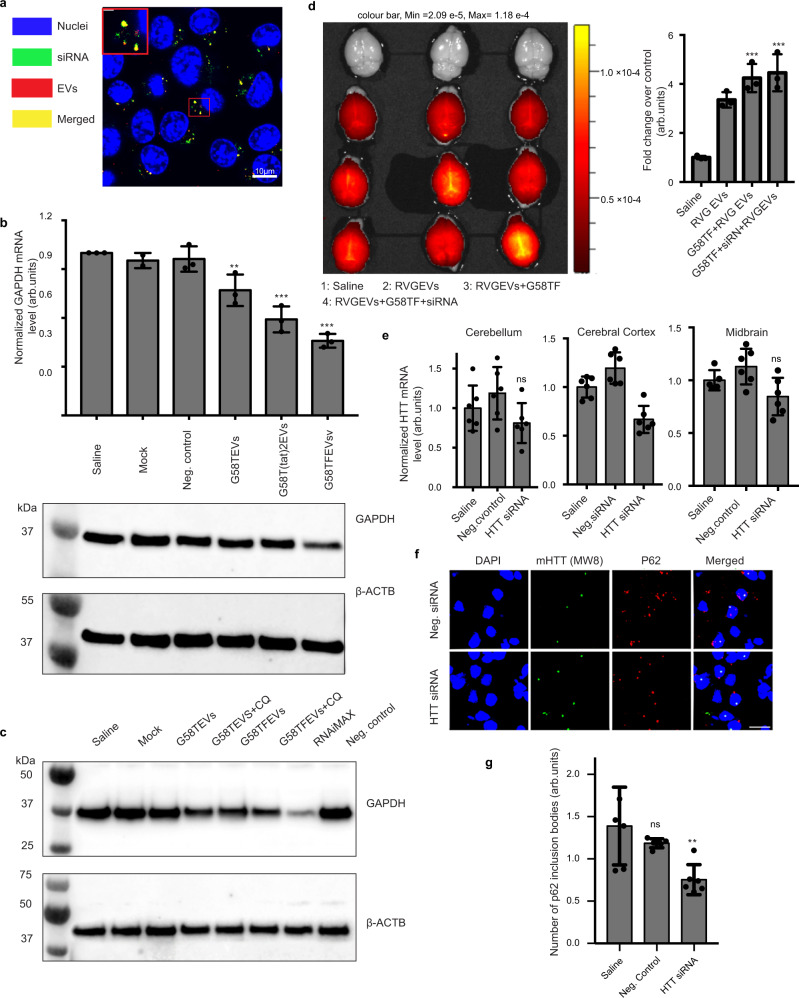
G58 peptide promotes EV-mediated siRNA delivery to the brain. **a** Confocal microscopy image of Neuro-2a cells after 4 h of incubation with siRNA-loaded G58T EVs. Nuclei of cells were stained with Hoechst 3342. EV surface proteins were labeled with Alexa fluor-633 (red) and siRNA was labeled with Cy3 dye (green). Inset is the magnified image (scale bar: 2 µm) of the marked region showing co-localization of EVs with siRNA (yellow spots). Scale bar:10 µm. The experiment was independently repeated four times. **b** Silencing of GAPDH gene in N2a cells by EVs engineered with G58T, G58T(tat)2 and G58TF proteins, respectively. Thirty nanomolar of GAPDH siRNA was loaded on to EVs, which were added to cells. Top histogram represents *GAPDH* mRNA level determined after 48 h of treatment, using probe-based RT-qPCR. Data were normalized with 18 S rRNA. Mock: G58TF-bound EVs alone, Neg. Control: G58TF EVs + non-targeting siRNA. Results shown as mean ± s.d (*n* = 3 independent biological experiment). Statistical differences were determined by one-way ANOVA, using Dunnett’s multiple comparisons test. During the analysis, saline group was used as control to determined statistical significance. **p = 0.0061, ***p = 0.0001. Bottom western blot shows GAPDH protein level in N2a cells after 72 h of treatment. The experiment was independently repeated three times. **c** Western blot showing effect of chloroquine (CQ) on silencing of GAPDH protein by G58T and G58TF EVs in N2a cells. (Mock: G58TF EVs alone, Neg. Control: G58TF EVs + non-targeting siRNA, RNAiMax: siRNA with lipofectamine RNAiMAX reagent. Thirty micromolar chloroquine was added to cells along with the EVs. The experiment was independently repeated two times. **d** In vivo fluorescence images of C57BL/6 mouse brains showing biodistribution of RVG-EVs, G58TF-RVG-EVs and G58T/siRNA-RVG-EVs. Images were taken after 4 h of systemic administration of EVs. Surface proteins of RVG-EVs were labeled with cy5.5-NHS fluorescent dye. Mice injected with saline were used as a negative control. Histogram at the right side shows quantification of the fluorescent signal from the brains of treated mice. (*n* = 3 mice). Results shown as mean ± s.d. Statistical differences were determined by one-way ANOVA, using Dunnett’s multiple comparisons test. **p = 0.0011, ***p = 0.0001. Animal group treated with saline was used as control to determine the *p*-value. **e** In vivo silencing of *Htt* gene in Q140 HD mouse model after systemic administration of G58TF EVs. Animals received four injections of EVs over four weeks. Seventy two hours after the last dose, animals were sacrificed and sections of brain were analyzed for *Htt* mRNA level, using probe-based RT-qPCR. Data were normalized with 18 S rRNA (*n* = 6 mice). Results are shown as mean ± s.d. Statistical differences were determined by one-way ANOVA, using Dunnett’s multiple comparisons test. ns non-significant, **P = 0.0012 when compared to control group (saline). **f** Immunohistochemistry of cortical regions from the treated animals. Images show mutant HTT protein level and p62-labeled inclusion bodies in the neurons of the cortex of *Htt* siRNA and Neg. siRNA-treated animals (Scale bar indicated 15 µm). **g** Histogram representing quantification of p62 inclusion bodies. Results are mean ± s.d, *n* = 8 slides chosen randomly from each group. Statistical differences were determined using one-way ANOVA (two tails) and post-hoc adjustment using Dunnett’s test. **P = 0.0031 when compared to saline group. Source data are provided as a Source Data file.

Given the physiological role of GAPDH in exosome biogenesis and its binding to CD63-positive human EVs, we reasoned that the binding of the GAPDH G58 peptide to the outer surface of EVs could be utilized as a specific, but generic, tool to attach therapeutic moieties to the surface of EVs. As a proof-of-principle study, we found that the G58T fusion bound siRNA with high efficiency, as evidenced by gel-shift assay and spectrofluorimetric analysis (~500–700 siRNA molecules per EV; Supplementary Fig. 7a and 7b). Moreover, bound siRNA was protected from degradation by RNase A (Supplementary Fig. 7c) Confocal microscopy of N2a cells treated with Alexa fluor-633-labelled G58T EVs loaded with Cy3-labelled siRNA revealed efficient uptake of complexes by the cells (Fig. 4a). Gene silencing assays using predesigned *Gapdh*-targeting siRNA, however, revealed low levels of gene silencing (~15%) in N2a cells treated with G58T EV siRNA. Co-localization studies using lysotracker dye suggested entrapment of the delivered siRNA in late-endosomes (Supplementary Fig. 7d and 7e). To overcome endosomal entrapment, we investigated the fusion of different endosomolytic peptides including TAT, HA2 and the arginine-rich peptide of flock house nodovirus (FHV) to the G58T protein. These peptides have previously been successfully used to enhance the release of drugs from late-endosomes^48,49^. Attachment of either two TAT peptides or the FHV peptide to the G58T protein (designated as G58T(tat)2 and G58TF respectively) resulted in significantly improved activity with ~35 and 60% silencing of endogenous genes (*GAPDH* and *HTT*) in the cells, respectively (Fig. 4b; Supplementary Fig. 7f). HA2 chimeric proteins could not be expressed due to toxicity of the peptide in the *E. coli* producer cells. Further treatment of cells with chloroquine, an endosomolytic molecule^50^, enhanced gene silencing efficiency to around 80%, consistent with the release of entrapped siRNA from late-endosomes (Fig. 4c; Supplementary Fig. 7g and 7h). Taken together, these results show highly efficient loading and delivery of siRNA into cells by G58T-functionalized EVs, resulting in potent gene silencing when combined with fusion of endosomolytic peptides to G58T protein.

### Silencing of *Htt* gene in the brain of Q140 mice after systemic administration of G58TF EVs/siRNA

To further assess the in vivo therapeutic potential of G58-engineered EVs, we investigated targeted delivery of siRNA into the mouse brain by co-expressing the RVG peptide on the EV surface. This peptide, which binds specifically to acetylcholine receptors, has been extensively used to deliver drugs into the brain^51^. Previous studies from our laboratory have shown that RVG increased brain accumulation of EVs without inducing toxicities in mice^52,53^. To determine whether the binding of G58TF (G58T with FHV domain) protein to the EV surface alters the biodistribution of RVG-EVs, we systemically injected C57BL/6 mice with RVG-EVs. Four hours after administration, ~1% of injected EVs were found in the brain, which is ~4.0 × 10^11^ EVs for a 20 g of mice. Similar results have been observed previously while studying biodistribution of EVs in different tissues of the mice^53^. Binding of either G58TF or G58TF/siRNA to RVG-EVs did not change their biodistribution (Fig. 4d), although, as anticipated, the majority of EVs were distributed to peripheral tissues, indicating rapid clearance of EVs from the blood (Supplementary Fig. 8).

To assess the silencing efficiency and therapeutic potential of G58TF/siRNA-RVG-EVs in the brain, we targeted silencing of the huntingtin gene (*Htt*) in the Huntington’s disease (HD) mouse model Q140^54^. HD is a neurodegenerative disease caused by mutation in the huntingtin gene that results in polyglutamine (poly Q) expansion in the protein, causing death of neurons^55^. The monogenic and dominant nature of the disease, and availability of HD mouse models provides a unique opportunity to develop and assess therapeutic potential of non-coding RNA in silencing expression of the mutant *Htt* gene^54^. In this study, using a mixture of siRNA targeting different regions of the *Htt* exon of Q140 mice, we systemically administered a total of four doses, once per week. Analysis of brain tissues revealed almost 40% silencing of the *Htt* gene in mouse brain cortex and a highly significant decrease of p62 inclusion bodies in the cortical neurons of the treated animals, an important phenotypic HD marker of disease progression (Fig. 4e, f). p62 is an important regulatory protein of selective autophagy, and a reduction in p62 aggregates in HD mouse models has previously been shown to restore HD-associated phenotypes^56^. However, for reasons that we currently do not understand, low levels of *Htt* silencing was observed in other parts of the brain, which is deserving of further investigation. Moreover, the slow progression of disease phenotypes in Q140 mice makes it difficult to determine morphological and phenotypic changes of HD symptoms in treated mice. Taken together, our study demonstrated a promising application of G58-coupled EVs in delivery of siRNA to brain with the potential for pre-clinical and clinical development.

## Discussion

EVs have considerable potential as bio-delivery vehicles, but our limited understanding of EV biogenesis and loading remains one of the obstacles in developing these natural signaling mediators as a therapeutic tool. Here, we demonstrate that endogenous GAPDH resides on the outer surface of eukaryotic EVs from multiple cell sources and binds to these vesicles via its phosphatidylserine-interacting domain. One function of this interaction is to promote clustering of exosomes, which appears to play an important role in Rab11-exosome biogenesis and organizing the large secretory compartments produced by *Drosophila* secondary cells. We find there are a substantial number of free GAPDH binding sites on the outer surface of human EVs, which can be harnessed for loading therapeutic siRNA onto EVs. Using this approach, we silenced the huntingtin gene in the brain of a Huntington’s disease mouse model after intravenous administration.

GAPDH is a multidimensional protein that is intimately involved in diverse physiological functions and human pathologies. The mechanisms by which these multiple functions are coordinately regulated remain largely elusive. Unlike other proteins, GAPDH is encoded by a single mRNA species in human cells. It is, therefore, believed that the multifunctional nature of the protein is generated by interaction with different cellular components and acquisition of distinct activities via post-translational modification in a range of subcellular locations^57^. Structural analysis of GAPDH identified NAD^+^-binding and catalytic domains of the protein^58^. In accordance with the earlier reports, we demonstrated that the PS-binding domain located within the NAD^+^ domain is responsible for association with the surface of EVs. An enzymatic assay confirmed that GAPDH bound to the EV surface is catalytically active, which is in contrast to earlier reports where GAPDH isolated from rabbit brain was found to induce fusion of synthetic liposomes but lacked glycolytic activity^37^. Catalytic activity of GAPDH has been reported to play a vital role in trafficking of vesicles along axonal segments of the nerve cells by providing constant supply of ATP to molecular motor proteins, but in this case, the GAPDH is located in the cytosol^59^. Whether post-translational modifications (PTM) of the protein have any role in modulating the binding to EVs remains to be determined. However, due to the limited capacity of bacterial cells to introduce PTM^60^, it appears that these modifications are not required for EV binding.

To address which domains and/or amino acid residues are involved in mediating binding of GAPDH to the EV surface, we developed several GAPDH mutants. Deletion of the catalytic domain did not affect binding of GAPDH’s NAD^+^ domain to EVs. Interestingly, mutation of two amino acid residues within the PS-binding domain of GAPDH significantly decreased binding of mutant GAPDH to EVs without affecting its glycolytic functions, corroborating earlier observations that neither neither β-NAD^+^ nor DL-glyceraldehyde-3-phosphate had a significant effect on binding of GAPDH to PS lipids of the liposomes, suggesting that PS-binding domain is not related to the NAD^+^ binding site (also known as Rossman fold) or catalytic domain^39^. These results further confirm our in vivo observations about non-enzymatic role of GAPDH in biogenesis of EVs.

GAPDH is one of the top five most common proteins associated with EVs^61^. Some studies have reported the presence of GAPDH within the EV lumen, but others have identified it on the EV’s outer surface^33,62^. Using a simple protease digestion assay to distinguish internal and external proteins, we found GAPDH exclusively on the outer surface of EVs. In one recent study, GAPDH was identified in non-vesicular aggregates from small EV preparations and was not pulled-down using immunocapture with magnetic beads coupled to CD63, employed in this analysis as a putative exosome marker^43^. However, we have confirmed that GAPDH is present on the outer surface of human HEK293F- and MSC-derived EVs using fluorescently labelled anti-GAPDH antibodies and high-resolution single EV IFCM, and that it co-associates with a subset of CD63-positive EVs. Whether other CD63-positive EVs are loaded with low levels of GAPDH that are below the detection limit of this method remains unclear. GAPDH was found to bind the EV surface via PS-binding domain. The limited proportion of vesicles that bear both GAPDH and CD63 molecules could be due to several reasons, including low affinity of the GAPDH antibody used in our experiments, binding of GAPDH to EVs that lack CD63 markers and alteration of EV markers due to overexpression of CD63-GFP on the EV surface.

Recently, it has been demonstrated that cytosolic GAPDH associates with ILVs in early and late-endosomes of human cells through the process of endosomal microautophagy, which involves the ESCRT machinery and HSC70 (Heat shock cognate 71 kDa) protein^34^. This GAPDH is secreted on exosomes and through its interaction with transferrin and lactoferrin can act as a sponge, which permits the uptake of free iron. Given these findings and our observation that exogenous GAPDH leads to EV clustering, we investigated the role of GAPDH in exosome biogenesis, using our unique *Drosophila* SC model in which clustered Rab11-exosome formation has recently been visualized in large endosomal compartments by fluorescence microscopy^44^. When overexpressed, human GAPDH trafficked into endosomal compartments of SCs and induced vesicle clustering both inside and outside of these cells, suggesting that the in vitro EV clustering activity is replicated in vivo. Furthermore, we found that *Drosophila* GAPDH2 has EV clustering activity in vitro and has important in vivo functions in SCs, since *GAPDH2* knockdown suppressed normal ILV clustering in Rab11-endosomes, reduced the proportion of ILV-generating compartments and decreased Rab11-exosome secretion. Similar effects were not observed with knockdown of other glycolytic enzymes, suggesting this is not the result of metabolic changes. Importantly, we have found that human cancer cell lines also produce Rab11-exosomes, particularly under nutrient stress conditions, so our observations are unlikely to be *Drosophila*-specific^44^. *GAPDH2* knockdown also affected the formation of a single large dense-core granule in Rab11 compartments, consistent with recent *ESCRT* knockdown experiments in SCs^47^, which suggest that exosome and DCG biogenesis are inter-dependent. Our current model is that clustered ILVs play a role in stabilizing a large DCG in SCs^46^, but this does not exclude the possibility that DCGs also affect ILV formation. The reduction in vesicle clustering caused by *GAPDH2* knockdown may also affect continued ILV biogenesis. Suppression of exosome secretion may be partly associated with the proposed roles of GAPDH in membrane fusion, which have been shown to regulate secretion of other DCG compartments^63^. Both *GAPDH1* and *GAPDH2* knockdown, but not knockdown of other glycolytic enzymes, affects Rab11-exosome secretion in SCs, suggesting that both of these proteins may work together in this specific process.

Indeed, clustered ILVs have been observed in other cell types in mammals and nematodes in the absence of DCGs and in specific pathological scenarios^36,64^. One common feature in these studies is that the MVBs involved may not be classical late-endosomes. Indeed, exosome clustering can be induced when the endolysosomal V-ATPase is inhibited, which may suggest a requirement for more neutral pH and has been suggested to modulate the range of exosome signaling^65^. In our analysis, the presence of two *Drosophila* GAPDH isoforms may have been critical in allowing us to define this enzyme’s specific roles in exosome clustering and biogenesis. By contrast, we found that the levels of knockdown of the single *GAPDH* gene in human cells were too variable and led to excessive toxicity (data not shown), preventing us from assessing the importance of this molecule in exosome and EV production in cultured human cells.

We have shown that the PS-binding domain (G58) can be used in fusion proteins to load cargos on to the surface of EVs, without the requirement to engineer cells for endogenous loading. Flow cytometry confirmed that most EVs that already carry surface GAPDH have many available sites for additional G58T fusion protein loading. The presence of RNA on the surface of EVs potentially exposes them to serum nucleases and immune cells, which could drastically reduce their therapeutic potential. However, a previous siRNA binding study using the dsRBD of TARBP2 protein has shown that this domain protects siRNA from degradation by nucleases and significantly reduces activation of the innate immune response to siRNA when compared to naked siRNA, consistent with our finding that G58TF/siRNA has functional effects in vivo^66^.

Uptake and gene silencing studies with G58T EVs revealed trafficking of these EVs to late-endosomes and lysosomes. The addition of chloroquine and/or G58TF peptide increased siRNA efficacy, presumably by favouring release from these compartments. Recently a detailed study has demonstrated that endocytosed EVs in large endosomal compartments traffic towards the endoplasmic reticulum and finally sort into late-endosomes^67^. However, how intravesicular or extravesicular exosomal cargo is transferred to the cytosol remains unresolved, although the ability of siRNAs located on either side of the EV membrane to induce gene knockdown indicates both routes are possible^15,16^.

Huntington’s (HD) disease is a progressive neurological disorder that is caused due to toxic gain of gene function. Currently, there are no successful drugs to cure the disease^68^. Recently, reducing the expression of mutant *HTT* gene using siRNA has emerged as a promising treatment^69^. In our experiments, although the majority of the EVs administered were rapidly cleared from each mouse, a small percentage of EVs (~1%) was found in the brain and was able to knockdown the *Htt* gene. The significant reduction of p62 aggregates achieved in the cortex of Q140 mice at this moderate level of *Htt* gene silencing highlights the therapeutic potential of our approach in the treatment of HD.

The knocked-in CAG repeat expansion in Q140 mice and the consistent expression of the mutant *Htt* gene and protein in the brain were important reasons to initially test the therapeutic potential of G58TF EVs in these mice. However, slow progression of disease symptoms (>12 months) in Q140 mice makes it difficult to rapidly assess the behavioural and phenotypic changes of disease pathology in this model. Therefore, further studies on the R6/1or R6/2 models, which demonstrate a much more aggressive phenotype than Q140 mice^70^, will be necessary to determine the clinical significance of *Htt* gene silencing by G58TF EVs. Furthermore, strategies to elevate levels of these EVs in the blood by expressing the CD47 receptor, which increases retention of circulating EVs^15^, and by optimization of the G58TF EV formulation should enhance the percentage of gene silencing in the brain for these experiments.

In summary, we have demonstrated in vitro and in vivo that GAPDH induces clustering of EVs, and shown that this activity plays a physiological role in clustering ILVs in endosomal compartments of SCs, which appears to impact on other intraluminal events, such as DCG biogenesis, in these compartments. More importantly, by engineering GAPDH chimeric proteins that have retained the G58 EV-binding domain, but do not induce EV clustering, we have developed a simple and highly robust method for therapeutic loading of RNA-based drugs on EVs for targeted drug delivery both in vitro and in vivo in pre-clinical disease models. The specific and strong affinity of the G58 peptide for EVs should permit loading of siRNAs onto crude EV preparations from different cell sources or even biofluids from patients. Furthermore, the modular design employed for the delivery of other types of cargoes, ranging from different types of nucleic acids including guide RNAs, to proteins and peptides, and for targeting EVs to other specific cells or tissues, thereby enhancing the therapeutic potential.

## Methods

### Isolation, purification and characterization of extracellular vesicles

Mesenchymal stem cells (MSCs), human embryonic kidney cells (HEK293T), human ovarian cancer cells (SKOVE-3), B-lymphoma cells (B16-F10) and HeLa cells were used to isolate EVs. All the cells were purchased from ATCC. Cells were grown in DMEM GlutaMax medium (ThermoFisher Scientific, UK), containing 10% fetal bovine serum and 1 × penicillin-streptomycin (PS, 100 × contains 5000 U/ml of penicillin and 5000 µg/ml streptomycin; Sigma). MSCs were grown in RPMI GlutaMax media (ThermoFisher Scientific) with 10% FBS and 1 × PS (Sigma). Cells were maintained at 37 °C with 5% CO_2_. For isolation of EVs, cells were seeded in 150 × 20 mm dishes (Star Labs, UK) at 10^6^ cells per dish in DMEM + 10% FBS media. Twenty four hours after seeding, the media of the cells was replaced with Opti-MEM reduced serum media (ThermoFisher Scientific, UK). Cells were incubated further for 48 h followed by collection of media in 50 ml falcon tubes (Sigma). To remove dead and floating cells, the media was centrifuged at 500 × *g* for 5 min. The supernatant was gently transferred into a fresh tube and centrifuged at 3000 × *g* for 20 min at 4 °C to pellet cell fragments and remaining cell debris. Purified media was concentrated by tangential ultrafiltration (TFF), using 100 kDa Vivaflow 50 R cartridges (Sartorius UK limited). Concentrated media was centrifuged at 10,000 × *g* for 30 min to remove aggregates and larger particles. The volume of media was further reduced to 2 ml, using Amicon ultra-15 centrifugal filter units of 100 kDa MWCO (Millipore). Finally, the media was passed through a Sepharose 4 fast flow gel-filtration column (GE Healthcare, 170149001), using the ÄKTA pure chromatography system (GE Healthcare). Purified EVs eluted from the column were used for the various biological assays. Typically, 500 ml of cell-culture media was used at the very beginning of the process and concentrated to 2 ml before loading to the column.

For animal experiments, large quantities of EVs were isolated using a hollow fibre bioreactor (FibreCell System, UK). 5 × 10^8^ MSCs or HEK293T cells were seeded into the extra-capillary space (ECS) of a hollow fibre bioreactor cartridge as per the guidelines of the manufacturer. The level of glucose in the media was measured on a daily basis to monitor cell growth. After 1 week of growth, extracellular material was harvested by gently flushing 25 ml of Opti-MEM media into the ECS from the left end port to collect extracellular material on right end port of the cartridge. Withdrawn medium was flushed back and forth between the syringes, through the ECS, four times to dislodge material collected within the fibre bundles. The resulting harvest was used for isolation of EVs using the differential centrifugation, ultrafiltration and size-exclusion chromatography methodology as described above.

### Nanoparticle tracking analysis

To measure size distribution and number of EVs, nanoparticle tracking analysis (NTA) was performed using the NS500 nanoparticles analyser (﻿NanoSight, Malvern, Worcestershire, UK)^71^. Dilutions of EVs ranging from 1:500 to 1:1000 in PBS were used to achieve a particle count of between 2 × 10^8^ and 2 × 10^9^ ml^−1^. During the recordings, a camera level of 12–14 was used. Using the script control function, three 30 s videos of each sample were recorded. After each video, a fresh sample was injected into the stage followed by a delay of 7 s to reduce turbulence of the flow. NTA2.3 software provided by the manufacturer was used to analyse and measure size and concentration of the EVs. All NTA measurements were carried out in triplicate.

### EV protease digestion assay

HEK293T and HeLa cells were seeded at a density of 8 × 10^6^ cells in 15 cm cell-culture dishes. After 24 h, cells were co-transfected with CD81-GFP and DC-LAMP-lactoferrin encoding plasmids and EVs were isolated 48 h later. Purified EVs were concentrated in Amicon ultra 2 ml centrifugal filters (Merck, UK) at 4 °C in table-top centrifuge at 3200 × *g*. Concentrated EVs were analysed by NTA to determine number of EVs. 1 × 10^12^ EVs in 30 µl of PBS buffer were added to PCR tubes. The tubes were divided into three groups as:

Group I: Normal EVs. In this group neither proteases nor denaturants were added.

Group II: Protease treated EVs. Here, 0.2 µg/µl of pronase (mixture of proteases, Sigma UK) was added to EVs.

Group III: Denatured EVs treated with proteases. In this group, EVs were denatured by adding 0.1% triton X-100 followed by heating EVs at 100 °C for 5 min. After EVs were cooled down to room temperature, 0.2 µg/µl of pronase was added.

Samples treated with Pronase were incubated at 37 °C at different timepoints, varying from 5 to 60 min. After the incubation, proteases were inactivated by adding a protease inhibitor (final concentration 10× from 100× stock, Cambridge Bioscience UK). The samples were further incubated at room temperature for 5 min to allow inactivation of proteases followed by addition of reducing lithium dodecyl sulfate sample loading buffer (0.5× final concentration, ThermoFisher Scientific). The samples were immediately transferred into PCR machine maintained at 70 °C for 10 min. Treated and non-treated EV samples were loaded into SDS-PAGE (Bolt 4–12%, Bis-Tris gels, ThermoFisher Scientific UK) for western blotting. For B16-F10, SKOV-3 and MSCs cells, alix protein was used as a positive control for proteins residing in the lumen of the vesicles.

### Electron microscopy

For electron microscopy (EM) analysis of EVs, samples were prepared as per the protocol described previously^72^. Briefly, 20 µl drops of purified EVs were added to parafilm and formvar-carbon-coated EM grids ﻿(Agar Scientific, Stansted, UK), which were floated on the top of EV droplets for 10 min with the coated side of the grid facing the drop. After the incubation, the grids were carefully transferred to PBS droplets for washing. Finally, the grids were placed briefly on water droplets to remove excess salt. For contrasting, grids were initially transferred ﻿to a 50 µl drop of uranyl oxalate solution, pH 7, for 5 min, followed by embedding in 2% uranyl acetate for 5 min. Next, the grids were carefully removed and excess of fluid was removed by gently ﻿pushing the grid sideways on Whatman no. 1 filter paper. The grids were air dried and visualized ﻿under a JEOL 1010 transmission electron microscope at 120 kV on an EFI Tecnai 12 TEM (JEOL, Peabody, MA, USA).

### Western blot

For western blotting, EV lysates corresponding to 5–10 µg of total protein were separated on 4–12% Bis-Tris plus gels (Invitrogen, ThermoFisher Scientific). Prior to loading, EV proteins were denatured by adding NuPAGE LDS Sample loading dye and reducing agent (ThermoFisher Scientific) to the EVs and heated at 70 °C for 10 min. Samples loaded on the gel were run at 150 V for 1 h in ice-cold NuPAGE MES running buffer. Proteins resolved on the gel were transferred onto 0.45 µm Immobilon-P, PVDF Membrane (Millipore), using a mini transfer blot cell (Bio-Rad). Membranes were blocked with SuperBlock T20 (TBS) Blocking Buffer (ThermoFisher Scientific) for 1 h at room temperature with gentle shaking. After blocking, primary antibodies diluted in the blocking buffer were added to the membrane and incubated overnight at 4 °C on a shaker. Details of the antibodies used for western blotting of EVs are given in Table S1. After the incubation, the membranes were washed three times for 5 min each, using 1× tris-buffered saline, pH 7.6 (TBST, 20 mm Tris base, 150 mM of NaCl and 0.1% Tween). After the final wash, membranes were incubated with horseradish peroxidase (HRP)-conjugated secondary antibodies for 1 h at room temperature with gentle shaking. Post-incubation, membranes were washed three times for 5 min each with 1× TBST buffer and developed for by chemiluminescence detection (GE healthcare, RPN2106). The Odyssey FC imaging system (LI-COR) was used to visualize the bands on the membrane. Image studio software provided with the system was used to analyse the data.

For western blotting of proteins from cells, ﻿radioimmunoprecipitation assay (RIPA) buffer (ThermoFisher Scientific) containing 1× protease inhibitor (Halt protease inhibitor, ThermoFisher Scientific) was added to cells and incubated on ice for 10 min. Lysates of the cells were passed through 26 G needle syringes several times and centrifuged at 12,000 × *g* for 20 min to remove genomic DNA aggregates and insoluble lipids. Cell supernatants corresponding to 5–10 µg of the total proteins were used for the western blotting.

### Purification of GAPDH from bacterial cells

The cDNA sequence of human GAPDH (OriGene, UK) was inserted into the pET-28b(+) vector (Novagen). Six residues of histidine were inserted at the N-terminus for purification by Ni-NTA chromatography and a FLAG-tag inserted at the C-terminus. The vector was transformed into BL21(DE3) competent *E. coli* cells (New

England Biolabs, UK) for expression. Sequences of *GAPDH* gene and primers used for cloning are given in Supplementary Table 6. 1 mM isopropyl β-D-1-thiogalactopyranoside (IPTG, Sigma) was used to induce expression of the gene in cells cultured at 37 °C. GAPDH protein was purified by Ni-NTA chromatography (Qiagen) under native conditions, using standard protocols recommended by the manufacturer. Ice-cold sodium-phosphate buffer, pH 8.0 (50 mM Na_2_HPO_4_/NaH_2_PO_4_, 10 mM Tris-Cl, 300 mM NaCl, 5 mM imidazole) was used during resuspension and lysis of the BL21(DE3) cells. Purified GAPDH protein was eluted with 250 mM imidazole in sodium-phosphate buffer, pH 6.0. Protein samples were dialyzed and passed through a Sephacryl s-200 HR column (GE Health Care) for further purification. The protein was analysed by SDS-PAGE, and the size of the protein was confirmed by mass spectrometry, using matrix-assisted laser desorption/ionization-time of flight-mass spectrometry (MALDI-TOF-MS).

### G58T, G58T(TAT)_2_ and G58TF chimeric protein

The DNA sequence of *GAPDH* gene, which encodes the PS-binding domain (designated as G58), was fused with the second domain of the human TAR RNA-binding protein (TARBP2, domain sequence was from UniProtKB; Sequence ID: Q15633). The DNA sequence corresponding to 58–100 amino acid of GAPDH protein (PS-binding domain lies between 70–90 amino acids) were selected during fusion with TARBP2 second domain and recombinant DNA was synthesized commercially (Integrated DNA technology, Belgium). Additional amino acid sequence of G58 peptide (amino acid sequence from 58 to 69) were chosen for stability of G58 domain while fusing with other domains of the chimeric protein the chimeric protein. The DNA sequence of TAT and arginine-rich peptide of flock house virus alpha capsid protein was custom synthesized (Integrated DNA Technology) and fused to the TARBP2 second domain by PCR cloning. The sequence of G58 peptide and arrangement of domains in different G58 chimeric proteins is described in Table S2 and Table S7. The DNA sequences of these chimeric proteins were cloned into the pET-28b(+) vector. G58T was expressed in BL2(DE3) cells, using similar conditions to those mentioned above. G58T(TAT)_2_ and G58TF were expressed in BL2(DE3) Rosetta (Novagen, Merk, USA). The cells were grown normally at 37 °C until OD_600_ reached between 0.5 and 0.6. At this point, 0.2 mM IPTG was added to the culture, and bacteria were grown at 21 °C for 10–12 h. All versions of G58 chimeric proteins were purified by Ni-NTA chromatography under denaturing conditions, using 6 M urea. Purified proteins were analysed by SDS-PAGE and MALDI-TOF-MS, as described above. Proteins were refolded by the dilution method. Briefly, proteins bound to Ni-NTA resin were eluted by 250 mM imidazole in sodium-phosphate buffer, containing 2 M urea. Eluted proteins were further diluted with an equal volume of ice-cold PBS containing 5% glycerol and 0.1 mM DTT. After 5 min of incubation, the protein was added dropwise to PBS (with constant stirring) to reduce the urea concentration to 0.5 M. After the dilution, aggregated proteins were removed by centrifugation at 30,000 × *g* for 20 min (Beckman Coulter, USA). Protein was concentrated using centrifugal spin filters (Millipore) and passed through a PD10 column (GE Healthcare, USA) to remove final traces of urea.

### Generation of GAPDH mutants and their interaction with EVs

Using the human GAPDH cDNA sequence as a template different types of GAPDH mutant were developed. For the G150T clone, the NAD^+^ binding domain of GAPDH was amplified and fused with the second domain of the TARBP2 protein. The DNA sequence was inserted into pET-28b(+) for expression in BL21(DE3) *E.coli* cells *as* described above. The protein was purified under denatured conditions using a Ni-NTA matrix followed by refolding of the protein by gradual dilution of urea concentration. Purified protein was isolated by gel-filtration column chromatography. To insert specific single amino acid mutant in wtGAPDH, two codons specifying Arginine 80 and Lysine 84 were replaced with alanine codons. The whole gene was synthesized as gBlock (integrated DNA technologies, Belgium) and inserted into pET-28b vector, using standard restriction digestion and ligation protocols. After confirming the sequence, mtGAPDH was expressed and purified from BL21(DE3) *E. coli* cells using Ni-NTA matrix under native conditions. Both an enzymatic assay and SDS-PAGE were used to characterize the protein.

For incubation of mtGAPDH with EVs, HEK293T EVs isolated either from a Bioreactor or by culturing HEK293T cells in optiMEM media were purified using TFF and gel-exclusion chromatography. 1.4 × 10^11^ EVs in 700 ul of PBS buffer were incubated with either wtGAPDH or mtGAPDH for 2 h at 4 °C. Excess of unbound protein was removed by size-exclusion chromatography. After elution, EVs were counted by Nanoparticle tracking analysis (NTA) using Nanosight NS500 (Malvern, UK). 3.0 × 10^8^ particles in 10 µl of PBS were taken from each sample for GAPDH enzymatic kinetic assay (KDalert GAPDH assay, ThermoFisher Scientific). For immunoblotting, 7.5 × 10^9^ EV particles were heated at 70 °C for 10 min after adding reducing SDS loading dye. Samples were loaded into precast 4–12% Bis-Tris gels (Invitrogen, ThermoFisher Scientific). Proteins resolved on SDS-PAGE were transferred to a PVD membrane that was incubated with monoclonal antibodies against the desired proteins. The membrane was developed with chemiluminescence (GE Health Care, US). Images and quantification of the gels were carried out with Odyssey FC imaging system (LI-COR).

### Binding of GAPDH and G58T chimeric proteins to extracellular vesicles

For exogenous binding of GAPDH to EVs, purified EVs ranging from 7.5 × 10^11^ particles/ml to 1 × 10^12^ particles/ml were used for incubation with GAPDH and G58 chimeric proteins. Proteins (5–20 nmol as required) were added to 1 pmol of EVs and incubated for 2 h at 4 °C on a rotor top. Unbound proteins were removed by gel filtration. Proteins were also added directly to concentrated cell-culture medium, containing fetal calf bovine serum for binding followed by gel filtration to remove the unbound proteins from the EVs. Binding of GAPDH proteins to the EV surface was monitored by absorbance at 280 nm (Akta pure, GE Healthcare), western blot, GAPDH activity assay and gel-shift assay.

### GAPDH activity assay

A fluorescence-based KDalert GAPDH assay (Invitrogen, Life Technologies) was used to measure enzymatic activity of GAPDH protein of extracellular vesicles. The assay measures the conversion of NAD^+^ to NADH in the presence of phosphate and glyceraldehyde-3-phosphate (G-3-P). Under the recommended assay conditions, the rate of NADH production is proportional to the amount of GAPDH enzyme present. A fixed number of EVs were added to the substrate in a 96-well plate. A fluorescent microplate reader (Clariostar Plus, BMG Labtech) was used to acquire data using the kinetic mode setting, with *λ*_ex_ = 560 nm and *λ*_em_ = 590 nm. Data were analyzed using MARS Data Analysis software (Clariostar Plus, BMG Labtech).

### Gel-shift assay

Binding of G58T, G58T(TAT)2 and G58TF EVs to siRNA was assessed by gel-shift assay. 20 pmol of siRNA was added to a fixed number of EVs in PBS buffer and incubated for 10 min at room temperature. After the incubation, complexes were loaded onto a 2% agarose gel stained with 0.5 µg/ml ethidium bromide for visualization under a UV-transilluminator. Binding of siRNA to EVs was determined by analyzing the shift of siRNA on the gel. siRNA bound to EVs remained trapped near the wells while free siRNA migrated freely in the agarose gel. Using NTA data and the gel-binding assay, the number of siRNAs bound to EVs was determined.

### RNase A protection assay

For the RNase A protection assay, 0.2 mg/ml of RNase A (Qiagen) was added to EV-siRNA complexes and incubated for 6 h at 37 °C. The enzyme was inactivated by adding an equal volume of hot SDS lysis solution (2% SDS, 16 mM EDTA) and incubated at 100 °C for 5 min. siRNA was isolated using TRIzol reagent (Invitrogen, Life Technologies) and analysed by agarose-gel electrophoresis.

### High-resolution single EV analysis by imaging flow cytometry

For single EV analysis experiments by Imaging Flow Cytometry (IFCM), EVs were isolated from HEK293 Freestyle suspension cells (ThermoFisher) and immortalized human cord blood-derived mesenchymal stromal cells (MSCs; Inscreenex) through ultrafiltration and bind-elute size-exclusion chromatography (BE-SEC). In brief, conditioned medium was pre-cleared by low-speed centrifugation (5 min at 700 × *g*, then 15 min at 2000 × *g*) and by filtration through 0.22 µm filters (Corning, cellulose acetate, low protein binding) before it was concentrated and diafiltrated with two volumes of PBS by tangential flow filtration (300 kDa MidiKros columns, 370 cm^2^ surface area, Spectrum Labs). EVs were further purified with BE-SEC columns (HiScreen Capto Core 700 column, GE Healthcare) and concentrated using Amicon Ultra-15 10 kDa molecular weight cut-off spin filters (Millipore). Particle concentrations were assessed by nanoparticle tracking analysis with a NanoSight NS500 instrument. Samples were diluted in PBS 0.2% human albumin (albunorm, Octapharma AB) to a final concentration of 1 × 10^12^ particles/ml before use. For control purposes, EVs tagged with mNeonGreen (neon GFP) fused to CD63 were prepared from HEK293 freestyle cells stably expressing CD63-mNeonGreen^73^. GAPDH expression and G58 peptide binding were studied on the single EV level by high-resolution IFCM (Amnis Cellstream, Luminex; equipped with 405, 488, 561 and 642 nm lasers) based on previously optimized settings and protocols with an Amnis ImagestreamX MkII instrument. In brief, 25 µl of EVs at a concentration of 1 × 10^12^ particles/ml were incubated overnight at 4 °C with 400 pmol AlexaFluor488 labelled G58 peptide and/or with AlexaFluor647-labelled rabbit anti human GAPDH antibodies (abcam, ab204480, clone EPR16884) or APC-labelled mouse anti human CD63 antibodies (Miltenyi Biotec, 130–100–182; clone H5C6) at a final concentration of 8 nM in v-bottom 96 wells (ThermoFisher Scientific). Samples were diluted 1:10,000 in PBS before acquisition using the plate reader of the Cellstream instrument with FSC turned off, SSC laser set to 40%, and all other lasers set to 100% of the maximum power. Small EVs were defined as SSC (low) by using neon GFP-tagged EVs as biological reference material and regions to quantify fluorescence-positive populations were set according to unstained samples and single-stained controls. Samples were acquired for 5 min at a flow rate of 3.66 µl/min (setting: slow) with CellStream software version 1.2.3 and analyzed with FlowJo Software version 10.5.0 (FlowJo, LLC). Dulbecco’s PBS pH 7.4 (Gibco) was used as sheath fluid.

### Confocal microscopy

To determine uptake of siRNA-loaded EVs into cells, N2a cells were seeded onto coverslips in 6-well tissue culture plates (Sigma). Surface proteins of EVs were labelled with Alexa Fluor-633 (Invitrogen, Life Technologies). siRNA^Cy3^ was custom synthesized (Integrated DNA Technologies). After 24 h of seeding cells, complexes of Alexa Fluor-633-labelled EVs and siRNA^Cy3^ (20 pmol) were added to the cells and incubated for 6 h. After the incubation, cells were washed with PBS containing 20 U/ml heparin sulphate (Sigma), stained with Hoechst 33258 (Molecular probes, Life technologies) and fixed with 4% paraformaldehyde (ThermoFisher scientific, UK). The coverslips were mounted on glass slides using Ibidi mounting medium (Ibidi, Germany) and visualized using a fluorescence laser confocal microscope, ×60 oil immersion objective lens (FV 1000, Olympus). The data were processed using FV100 software.

### In vitro gene silencing by G58-modified extracellular vesicles

For silencing of genes in N2a cells and HeLa cells, G58T, G58T(TAT)2 and G58TF proteins were incubated with either MSC- or HEK293T-derived EVs. After binding, purified EVs were analyzed for siRNA binding by gel-shift assay, to determine the amount of EVs needed to completely bind a given amount of siRNA. Using this ratio, EVs with bound siRNAs were added to the cells in complete DMEM GlutaMax medium. Details of the siRNA used for gene silencing experiment are given in Table S3. After 24 h of incubation, medium was changed and cells were further incubated. For mRNA analysis, cells were harvested after 48 h of treatment, using TRIzol (Invitrogen, Life Technologies). Two hundred and fifty nanograms of total RNA was used for reverse transcription PCR, using the PrimeScript reverse transcriptase kit (Takara, Japan). Probe-based reverse transcriptase-quantitative PCR (RT-qPCR) was used to assess quantities of *GAPDH* and *Htt* mRNA. 18 S rRNA and Hypoxanthine-guanine phosphoribosyl transferase (*HPRT*) mRNA was used as reference genes for normalization. PCR efficiencies were determined using LinRegPCR. Data were analyzed using the ﻿Pfaffl method^74^. Details of RT-qPCR assays are provided in Table S4. For protein analysis, cells were harvested 72 h post treatment and western blotting performed as described above.

### Animal experiments

#### Biodistribution of extracellular vesicles

All animal experiments were performed in compliance with the Animals (Scientific Procedures) Act 1986, revised 2012 (ASPA, UK). Six-week-old female C57BL/6 mice were randomly assigned into four groups (*n* = 3). HEK293T cells stably transfected with RVG-LAMP-2B protein were seeded into 15 × 1.2 cm tissue culture plates for isolation of EVs. Immunoblotting of EVs and HEK293T cell lysates was carried out to assess expression of RVG peptide on surface of EVs. RVG-expressing EVs were labeled with sulpho-Cy5.5-NHS ester dyes (Abcam, ab235032). Briefly, 2 × 10^12^ EVs in 1 ml PBS buffer were incubated with 100 µmol of sulpho-Cy5.5-NHS ester, which was prepared in DMSO as a 10 mM stock. EVs were incubated at room temperature for 1 h with gentle shaking. After 1 h of incubation, G58TF protein was added to EVs and incubated for 2 h at 4 °C. Excess dye and protein was removed by passing the EVs through a gel-filtration column. After concentrating EVs, doses of EVs were given to animals as described in Table S5. After 4 h of administration, animals were sacrificed, organs harvested and Cy5.5 fluorescence visualized using the in vivo animal imaging system (IVIS, Perkin Elmer). Data were analyzed using the IVIS software.

#### Silencing of huntingtin gene *Htt* gene in Q140 Huntington disease model animals

Q140 mice were used to assess mutant and wild *Htt* gene silencing by intravenous administration of siRNA-loaded EVs. In the Q140 mouse model, exon 1 of mouse *Htt* is humanized and contains 140 CAG repeats. The mice have a slow progression of disease phenotype, which starts to appear at the age of 6 months. One-year-old Q140 mice were assigned to saline, negative siRNA control, and *Htt* siRNA treatment groups (*n* = 6). Saline group was used as a control to assess levels of *Htt* mRNA after administering EVs loaded with either negative siRNA (Negative group) or a mixture of *Htt* siRNA (treatment group). RVG-EVs bound to G58TF protein were used for the *Htt* silencing experiment. EV doses were calculated based on 0.5 mg/kg siRNA dosage regimen. The number of EVs needed to bind a given amount of siRNA were calculated by gel-shift assay. One hundred fifty to two hundred microlitres of EVs were administered intravenously. A total of four doses were given to animals at weekly intervals. Seventy two hours after the last dose, animals were euthanized and the various sections of the brain were analyzed for *Htt* mRNA and protein expression. For determining the level of HTT protein in the brain tissues, Agarose-gel electrophoresis for resolving aggregates (AGERA) was carried out to detect mutant HTT protein aggregates. However, we could not analyze the immunoblot due to high background. Immunohistochemistry of the cortex regions of the brain was carried out to determine the level of mutant HTT protein aggregates and p62 inclusion bodies.

#### *Drosophila* stocks and genetics

Flies were reared at 25 °C in vials containing standard cornmeal agar medium (12.5 g agar, 75 g cornmeal, 93 g glucose, 31.5 g inactivated yeast, 8.6 g potassium sodium tartrate, 0.7 g calcium, and 2.5 g Nipagen (dissolved in 12 ml ethanol) per litre. They were transferred onto fresh food every 2 days. No additional dried yeast was added to the vials. Temperature-controlled, SC-specific expression of *UAS-CD63-GFP* and *UAS-Btl-GFP* was achieved by combining these transgenes with the specific driver, *dsx-GAL4* and the temperature-sensitive, ubiquitously expressed repressor *tubulin-GAL80*^*ts*^. Newly enclosed virgin adult males were transferred to 29 °C to induce post-developmental SC-specific expression. For overexpression and knockdown experiments, the same strategy was employed, but in the presence of a UAS transgene or the *YFP-Rab11* gene trap. Six-day-old adult virgin males were used throughout this study to ensure that age- and mating-dependent effects on SC biology were mitigated^44,45^.

#### Preparation of accessory glands for live imaging and exosome secretion analysis

Adult male flies were anaesthetized using CO_2_. The abdomens were removed from anaesthetized flies and submerged in PBS (Gibco). The whole male reproductive tract was carefully pulled out of the body cavity. The accessory glands were isolated by separation of the ejaculatory bulb from the external genitalia, fat tissues and gut. Finally, the testes were removed by scission close to the seminal vesicles, as they often fold over the accessory glands, obscuring imaging.

The isolated accessory glands were transferred to a 9-spot depression glass plate (Corning PYREX) containing ice-cold PBS and kept on ice until sufficient numbers had been obtained. They were then stained with ice-cold 500 nM LysoTracker Red DND-99 (Invitrogen) (1:1000) in 1 × PBS for 5 min. Finally, the glands were rinsed in ice-cold PBS before being mounted onto high precision microscope cover glasses. A custom-built holder secured the cover glasses in place during imaging by wide-field microscopy. To avoid dehydration and hypoxia, the glands were carefully maintained in a small drop of PBS, surrounded by 10 S Voltalef (VWR Chemicals), an oxygen-diffusible hydrocarbon oil, and kept stably in place by the application of a small cover glass (VWR).

#### Imaging of *Drosophila* secondary cells

For wide-field imaging, living SCs were imaged using a DeltaVision Elite wide-field fluorescence deconvolution microscope (GE Healthcare Life Sciences) equipped with a ×100, NA 1.4 UPlanSApo oil objective lens (Olympus), and a Cool SNAP HQ2 CCD camera (Photometrics). The images acquired were typically z-stacks spanning 8–12 µm depth with a z-distance of 0.2 µm. Images were subsequently deconvolved using the Resolve 3D-constrained iterative deconvolution algorithm within the softWoRx 5.5 software package (GE Healthcare Life Sciences).

Confocal images of accessory glands were acquired by using LSM 880 laser scanning confocal microscope equipped with a ×63, NA 1.4 Plan APO oil DIC objective (Carl Zeiss). RI 1.514 objective immersion oil (Carl Zeiss) was employed.

#### *Drosophila* exosome secretion assay

Virgin six-day-old males of each genotype were dissected in 4% paraformaldehyde (Sigma–Aldrich) dissolved in PBS. The glands were left in 4% paraformaldehyde for 15 min to preserve the luminal contents before being washed in PBT (PBS containing 0.3% Triton X-100, Sigma–Aldrich) for 5 min. Glands were rinsed with PBS and then mounted onto SuperFrost Plus glass slides (VWR), removing excess liquid using a Gilson pipette, and finally immersed in a drop of Vectashield with DAPI (Vector Laboratories) for imaging by confocal microscopy.

Exosome secretion was measured by sampling within the central third of each gland. Identical microscope settings and equipment were used throughout. At each sampling location, ten different z-planes, spaced by 1 μm and at a distance from any SCs, were analysed.

The automated analysis of exosome secretion by SCs was performed using ImageJ2, distributed by Fiji. The ‘Noise>Despeckle’ function was first used to remove background noise, followed by conversion to a binary image. The number of fluorescent particles was assessed using the ‘Analyse Particles’ function. The average number of particles for all stacks was then quantified and compared to controls.

#### Analysis of ILV content in dense-core granule compartments

Living SCs from 6-day-old males of each genotype were imaged using identical settings by wide-field microscopy. The total number of DCG compartments that contained clustered fluorescently labelled puncta was counted in each cell, using a full z-stack of the epithelium. Three individual SCs were analysed from each of ten to fifteen glands.

#### Dense-core granule compartment analysis

Dense-core granule compartment numbers were manually quantified in ImageJ2 by using complete z-stacks of individual cells acquired with differential interference contrast (DIC) microscopy on the wide-field microscope. The morphology and position of DCGs were also analysed, and each compartment was scored for the presence of a single spherical DCG, an abnormally shaped DCG or multiple small fragmented DCGs. Three individual SCs per gland from each of ten to fifteen glands were used.

### Statistical analysis

All data are representative from at least two to three independent experiments. The distribution of residuals was tested for normality using Q–Q plots and the appropriate statistical test was applied. For quantification of western blots, Image Studio Lite (Li-Cor) software was used. Adobe Photoshop CS4 software was used to crop and arrange the western blotting, confocal microscopy and electron microscopy figures. Nonparametric one-way ANOVA, using Bartlett’s and Brown-Forsythe test, was used to calculate F and *P*-values. For mouse experiments, data were plotted and analysed, using multivariant (two-tail) ANOVA. For SC analysis and exosome biogenesis, the Kruskal–Wallis test was employed followed by Dunn’s test to compare individual control and experimental datasets. All data analyses and statistics were conducted using GraphPad Prism v8.0 (GraphPad Software Inc., La Jolla, CA, USA).

### Reporting summary

Further information on research design is available in the Nature Research Reporting Summary linked to this article.

## Supplementary information

Supplementary Information

## Data Availability

Source data accompanies this manuscript. The imaging data are available from the author upon request. The remaining data are available within the Article and Supplementary Information. Source data are provided with this paper.

## Source data

Source Data

## References

[1] Raposo G, Stahl PD (2019). Extracellular vesicles: a new communication paradigm?. Nat. Rev. Mol. Cell Biol..

[2] Maas SLN, Breakefield XO, Weaver AM (2017). Extracellular vesicles: unique intercellular delivery Vehicles. Trends Cell Biol..

[3] Willms E, Cabanas C, Mager I, Wood MJA, Vader P (2018). Extracellular vesicle heterogeneity: subpopulations, isolation techniques, and diverse functions in cancer progression. Front. Immunol..

[4] Iraci N, Leonardi T, Gessler F, Vega B, Pluchino S (2016). Focus on extracellular vesicles: physiological role and signalling properties of extracellular membrane vesicles. Int. J. Mol. Sci..

[5] Robbins PD, Morelli AE (2014). Regulation of immune responses by extracellular vesicles. Nat. Rev. Immunol..

[6] Zhang H (2017). Exosome-delivered EGFR regulates liver microenvironment to promote gastric cancer liver metastasis. Nat. Commun..

[7] Hoshino A (2015). Tumour exosome integrins determine organotropic metastasis. Nature.

[8] Varcianna A (2019). Micro-RNAs secreted through astrocyte-derived extracellular vesicles cause neuronal network degeneration in C9orf72 ALS. EBioMedicine.

[9] Wiklander, O. P. B., Brennan, M. A., Lotvall, J., Breakefield, X. O. & El Andaloussi, S. Advances in therapeutic applications of extracellular vesicles. *Sci. Transl. Med*. **11**, eaav8521 (2019). 10.1126/scitranslmed.aav8521 PMC7104415 31092696

[10] Zhang H (2018). Identification of distinct nanoparticles and subsets of extracellular vesicles by asymmetric flow field-flow fractionation. Nat. Cell Biol..

[11] Baietti MF (2012). Syndecan-syntenin-ALIX regulates the biogenesis of exosomes. Nat. Cell Biol..

[12] Trajkovic K (2008). Ceramide triggers budding of exosome vesicles into multivesicular endosomes. Science.

[13] ELA S, Mager I, Breakefield XO, Wood MJ (2013). Extracellular vesicles: biology and emerging therapeutic opportunities. Nat. Rev. Drug Discov..

[14] Johnsen KB (2014). A comprehensive overview of exosomes as drug delivery vehicles—endogenous nanocarriers for targeted cancer therapy. Biochim. Biophys. Acta.

[15] Kamerkar S (2017). Exosomes facilitate therapeutic targeting of oncogenic KRAS in pancreatic cancer. Nature.

[16] Gao X (2018). Anchor peptide captures, targets, and loads exosomes of diverse origins for diagnostics and therapy. Sci. Transl. Med.

[17] Elsharkasy OM (2020). Extracellular vesicles as drug delivery systems: why and how?. Adv. Drug Deliv. Rev.

[18] Tang TT, Lv LL, Lan HY, Liu BC (2019). Extracellular vesicles: opportunities and challenges for the treatment of renal diseases. Front. Physiol..

[19] Colombo M, Raposo G, Thery C (2014). Biogenesis, secretion, and intercellular interactions of exosomes and other extracellular vesicles. Annu. Rev. Cell Dev. Biol..

[20] Kooijmans SAA (2013). Electroporation-induced siRNA precipitation obscures the efficiency of siRNA loading into extracellular vesicles. J. Control Release.

[21] Smyth T (2014). Surface functionalization of exosomes using click chemistry. Bioconjug Chem..

[22] Sutaria DS, Badawi M, Phelps MA, Schmittgen TD (2017). Achieving the promise of therapeutic extracellular vesicles: the devil is in details of therapeutic loading. Pharm. Res..

[23] Sirover MA (1999). New insights into an old protein: the functional diversity of mammalian glyceraldehyde-3-phosphate dehydrogenase. Biochim. Biophys. Acta.

[24] Chang CH (2013). Posttranscriptional control of T cell effector function by aerobic glycolysis. Cell.

[25] Sundararaj KP (2004). Rapid shortening of telomere length in response to ceramide involves the inhibition of telomere binding activity of nuclear glyceraldehyde-3-phosphate dehydrogenase. J. Biol. Chem..

[26] Hara MR (2005). S-nitrosylated GAPDH initiates apoptotic cell death by nuclear translocation following Siah1 binding. Nat. Cell Biol..

[27] Tristan C, Shahani N, Sedlak TW, Sawa A (2011). The diverse functions of GAPDH: views from different subcellular compartments. Cell Signal.

[28] Jacquin MA (2013). GAPDH binds to active Akt, leading to Bcl-xL increase and escape from caspase-independent cell death. Cell Death Differ..

[29] Colell A (2007). GAPDH and autophagy preserve survival after apoptotic cytochrome c release in the absence of caspase activation. Cell.

[30] Butera G (2019). Regulation of autophagy by nuclear GAPDH and its aggregates in cancer and neurodegenerative disorders. Int. J. Mol. Sci..

[31] Hwang S, Disatnik MH, Mochly-Rosen D (2015). Impaired GAPDH-induced mitophagy contributes to the pathology of Huntington’s disease. EMBO Mol. Med.

[32] Zhang JY (2015). Critical protein GAPDH and its regulatory mechanisms in cancer cells. Cancer Biol. Med.

[33] Malhotra H (2016). Exosomes: tunable nano vehicles for macromolecular delivery of transferrin and lactoferrin to specific intracellular compartment. J. Biomed. Nanotechnol..

[34] Chauhan AS (2019). Trafficking of a multifunctional protein by endosomal microautophagy: linking two independent unconventional secretory pathways. FASEB J..

[35] Fillebeen C (1999). Receptor-mediated transcytosis of lactoferrin through the blood-brain barrier. J. Biol. Chem..

[36] Frankel EB (2017). Ist1 regulates ESCRT-III assembly and function during multivesicular endosome biogenesis in Caenorhabditis elegans embryos. Nat. Commun..

[37] Glaser PE, Gross RW (1995). Rapid plasmenylethanolamine-selective fusion of membrane bilayers catalyzed by an isoform of glyceraldehyde-3-phosphate dehydrogenase: discrimination between glycolytic and fusogenic roles of individual isoforms. Biochemistry.

[38] Nakagawa T (2003). Participation of a fusogenic protein, glyceraldehyde-3-phosphate dehydrogenase, in nuclear membrane assembly. J. Biol. Chem..

[39] Kaneda M, Takeuchi K, Inoue K, Umeda M (1997). Localization of the phosphatidylserine-binding site of glyceraldehyde-3-phosphate dehydrogenase responsible for membrane fusion. J. Biochem..

[40] Takahashi Y, Nishikawa M, Takakura Y (2017). In vivo tracking of extracellular vesicles in mice using fusion protein comprising lactadherin and gaussia luciferase. Methods Mol. Biol..

[41] Sirover MA (2014). Structural analysis of glyceraldehyde-3-phosphate dehydrogenase functional diversity. Int. J. Biochem. Cell Biol..

[42] Gorgens A (2019). Optimisation of imaging flow cytometry for the analysis of single extracellular vesicles by using fluorescence-tagged vesicles as biological reference material. J. Extracell. Vesicles.

[43] Jeppesen DK (2019). Reassessment of exosome composition. Cell.

[44] Fan, S. J. et al. Glutamine deprivation regulates the origin and function of cancer cell exosomes. *EMBO J* **39**, 0261–4189 (2020). 10.15252/embj.2019103009 PMC7429491 32720716

[45] Corrigan L (2014). BMP-regulated exosomes from Drosophila male reproductive glands reprogram female behavior. J. Cell Biol..

[46] Sun XH, Tso JY, Lis J, Wu R (1988). Differential regulation of the two glyceraldehyde-3-phosphate dehydrogenase genes during Drosophila development. Mol. Cell Biol..

[47] Marie, P. P. et. al. Accessory ESCRT-III proteins selectively regulate Rab11-exosome biogenesis in Drosophila secondary cells. *bioRxiv* 2020.06.18.158725; 10.1101/2020.06.18.158725.

[48] Wadia JS, Stan RV, Dowdy SF (2004). Transducible TAT-HA fusogenic peptide enhances escape of TAT-fusion proteins after lipid raft macropinocytosis. Nat. Med.

[49] Nakase I (2009). Cell-surface accumulation of flock house virus-derived peptide leads to efficient internalization via macropinocytosis. Mol. Ther..

[50] Hou KK, Pan H, Ratner L, Schlesinger PH, Wickline SA (2013). Mechanisms of nanoparticle-mediated siRNA transfection by melittin-derived peptides. ACS Nano.

[51] Huey R, Hawthorne S, McCarron P (2017). The potential use of rabies virus glycoprotein-derived peptides to facilitate drug delivery into the central nervous system: a mini review. J. Drug Target.

[52] Alvarez-Erviti L (2011). Delivery of siRNA to the mouse brain by systemic injection of targeted exosomes. Nat. Biotechnol..

[53] Wiklander OP (2015). Extracellular vesicle in vivo biodistribution is determined by cell source, route of administration and targeting. J. Extracell. Vesicles.

[54] Menalled LB, Sison JD, Dragatsis I, Zeitlin S, Chesselet MF (2003). Time course of early motor and neuropathological anomalies in a knock-in mouse model of Huntington’s disease with 140 CAG repeats. J. Comp. Neurol..

[55] Glorioso JC, Cohen JB, Carlisle DL, Munoz-Sanjuan I, Friedlander RM (2015). Moving toward a gene therapy for Huntington’s disease. Gene Ther..

[56] Kurosawa M (2015). Depletion of p62 reduces nuclear inclusions and paradoxically ameliorates disease phenotypes in Huntington’s model mice. Hum. Mol. Genet..

[57] Sirover MA (2012). Subcellular dynamics of multifunctional protein regulation: mechanisms of GAPDH intracellular translocation. J. Cell Biochem..

[58] White MR (2015). A dimer interface mutation in glyceraldehyde-3-phosphate dehydrogenase regulates its binding to AU-rich RNA. J. Biol. Chem..

[59] Zala D (2013). Vesicular glycolysis provides on-board energy for fast axonal transport. Cell.

[60] Tokmakov AA (2012). Multiple post-translational modifications affect heterologous protein synthesis. J. Biol. Chem..

[61] Kalra, H. et al. Vesiclepedia: A Compendium for Extracellular Vesicles with Continuous Community Annotation. *PLOS Biology* **10**, e1001450 (2012). 10.1371/journal.pbio.1001450 PMC3525526 23271954

[62] Jella KK (2016). Exosomal GAPDH from proximal tubule cells regulate ENaC activity. PLoS ONE.

[63] Han X, Ramanadham S, Turk J, Gross RW (1998). Reconstitution of membrane fusion between pancreatic islet secretory granules and plasma membranes: catalysis by a protein constituent recognized by monoclonal antibodies directed against glyceraldehyde-3-phosphate dehydrogenase. Biochim. Biophys. Acta.

[64] Muratori C (2009). Massive secretion by T cells is caused by HIV Nef in infected cells and by Nef transfer to bystander cells. Cell Host Microbe.

[65] Edgar, J. R., Manna, P. T., Nishimura, S., Banting, G. & Robinson, M. S. Tetherin is an exosomal tether. *Elife* **5**, 17180 (2016). 10.7554/eLife.17180 PMC5033606 27657169

[66] Dar GH, Gopal V, Rao M (2015). Conformation-dependent binding and tumor-targeted delivery of siRNA by a designed TRBP2: affibody fusion protein. Nanomedicine.

[67] Heusermann W (2016). Exosomes surf on filopodia to enter cells at endocytic hot spots, traffic within endosomes, and are targeted to the ER. J. Cell Biol..

[68] Ross CA (2014). Huntington disease: natural history, biomarkers and prospects for therapeutics. Nat. Rev. Neurol..

[69] Harper SQ (2005). RNA interference improves motor and neuropathological abnormalities in a Huntington’s disease mouse model. Proc. Natl Acad. Sci. USA.

[70] Lerner RP, Trejo Martinez Ldel C, Zhu C, Chesselet MF, Hickey MA (2012). Striatal atrophy and dendritic alterations in a knock-in mouse model of Huntington’s disease. Brain Res. Bull..

[71] Nordin JZ (2015). Ultrafiltration with size-exclusion liquid chromatography for high yield isolation of extracellular vesicles preserving intact biophysical and functional properties. Nanomedicine.

[72] Thery C, Amigorena S, Raposo G, Clayton A (2006). Isolation and characterization of exosomes from cell culture supernatants and biological fluids. Curr. Protoc. Cell Biol..

[73] Corso G (2017). Reproducible and scalable purification of extracellular vesicles using combined bind-elute and size exclusion chromatography. Sci. Rep..

[74] Roberts TC, Coenen-Stass AM, Betts CA, Wood MJ (2014). Detection and quantification of extracellular microRNAs in murine biofluids. Biol. Proced. Online.

