# Author Correction: GAPDH controls extracellular vesicle biogenesis and enhances the therapeutic potential of EV mediated siRNA delivery to the brain

**DOI:** 10.1038/s41467-021-27700-y

**Published:** 2021-12-16

**Authors:** Ghulam Hassan Dar, Cláudia C. Mendes, Wei-Li Kuan, Alfina A. Speciale, Mariana Conceição, André Görgens, Inna Uliyakina, Miguel J. Lobo, Wooi F. Lim, Samir EL Andaloussi, Imre Mäger, Thomas C. Roberts, Roger A. Barker, Deborah C. I. Goberdhan, Clive Wilson, Matthew J. A. Wood

**Affiliations:** 1 Department of Paediatrics, University of Oxford, Oxford, OX1 3QX UK; 2 Department of Physiology, Anatomy and Genetics, University of Oxford, Oxford, OX1 3QX UK; 3 John van Geest Centre for Brain Repair, Department of Clinical Neurosciences, University of Cambridge, Cambridge, CB2 0PY UK; 4 Department of Laboratory Medicine, Clinical Research Center, Karolinska Institutet, 14186 Stockholme, Sweden; 5 MDUK Oxford Neuromuscular Centre, University of Oxford, Oxford, OX2 9DU UK; 6 Oxford-Harrington Rare Disease Centre, University of Oxford, Oxford, OX2 9DU UK

**Keywords:** RNAi therapy, Extracellular signalling molecules

Correction to: *Nature Communications* 10.1038/s41467-021-27056-3, published online 18 November 2021.

The original version of this Article contained an error in Fig. 1d. In Fig. 1d the labelling of the EVs and cell lysate in the bottom panel was inadvertently switched. This error has been corrected in the PDF and HTML versions of the Article.

