# Supplementary material for: GAPDH controls extracellular vesicle biogenesis and enhances the therapeutic potential of EV mediated siRNA delivery to the brain
Source: Nat Commun. 2021 Nov 18;12:6666. doi: 10.1038/s41467-021-27056-3 (PMC8602309; doi:10.1038/s41467-021-27056-3)
Supplement: Supplementary file 1 — Supplementary Information [file 41467_2021_27056_MOESM1_ESM.pdf]

Supplementary Information

GAPDH controls extracellular vesicle biogenesis and enhances therapeutic potential of EV-mediated siRNA delivery to brain

Ghulam Hassan Dar[1], Cláudia C. Mendes[2], Wei-Li Kuan[3], Alfina A. Speciale[1], Mariana Conceição[1], André Görgens[4], Inna Uliyakina[2], Miguel J. Lobo[2], Wooi F. Lim[1], Anja Krueger[2], Samir EL Andaloussi[4], Imre Mäger[1], Thomas C. Roberts[1,5], Roger A. Barker[3], Deborah C. I. Goberdhan[2], Clive Wilson[2*], Matthew J.A. Wood[1,5,6*]

1. Department of Paediatrics, University of Oxford, OX1 3QX, Oxford, UK
2. Department of Physiology, Anatomy and Genetics, University of Oxford, OX1 3QX, Oxford, UK
3. Centre for Brain Repair, Department of Clinical Neurosciences, University of Cambridge, CB2 0PY, Cambridge, UK
4. Department of Laboratory Medicine, Karolinska Institute, 14186, Sweden
5. MDUK Oxford Neuromuscular Centre, University of Oxford, OX2 9DU, Oxford, UK
6. Oxford Harrington Rare Disease Centre, University of Oxford, OX2 9DU, Oxford, UK

[*]Email: matthew.wood@paediatrics.ox.ac.uk, clive.wilson@dpag.ox.ac.uk

Table of Contents

Supplementary Table 1 2
Supplementary Table 2 2
Supplementary Table 3...3
Supplementary Table 4 3
Supplementary Table 5 4
Supplementary Table 6 4
Supplementary Table 7.. 5
Supplementary Figure 1 6
Supplementary Figure 2 8
Supplementary Figure 3 9
Supplementary Figure 4 11
Supplementary Figure 5 14
Supplementary Figure 6 16
Supplementary Figure 7 18
Supplementary Figure 8 19
Supplementary Figure 9..20

Antibodies	Source	Catalogue number	Lot number	Dilution
Alix (1A12)	Santa Cruz Biotechnology,	sc-53540	H1518	1:500
CD81	Santa Cruz Biotechnology,	sc-166029	-	1:500
GAPDH	Sigma,	G9545-100µl	106M4851V	1:1,000
TARBP	Abcam,	ab72110	GR254409-2	1:1,000
cMyc [9E10]	Abcam,	ab32	GR255064-3	1:1,000
anti-Flag®M2	Sigma,	F3165-2MG	SLBN8915V	1:1,000
GFP [6AT316]	Abcam,	ab38689	GR216643-2	1:1,000
LAMP2B	Abcam,	ab18529	GR258631-1	1:1,000
Lactoferrin [EPR4337]	Abcam,	ab109216	YH031005C	1:1,000
Beta Actin	ThermoFisher Scientific,	MA5-15739	RC230092	1:2,000
Anti-Rabbit IgG-HRP	Sigma,	A0545-1mL	065M4769V	1:2,000
Anti-Mouse IgG-HRP	Sigma,	A5278-1mL	SLBN3514V	1:5,000
MMP-2(2c1)	Santa Cruz Biotechnology	, sc-13594	B1418	1:500

Supplementary Table 1: List of western blot primary antibodies used in this study.

**Constructs**
**G58T**
His_6-G58 peptide-(Gly_4Ser)-TARBP2 second domain
**G58T(tat)**
His_6-G58 peptide-(Gly_4Ser)-TARBP2 second domain-(PKR linker)-(TAT)-(TAT)
**G58TF**
His_6-G58 peptide-(Gly_4Ser)-TARBP2 second domain-(PKR linker)-(FHV peptide)
**Peptide Sequences**
**PKR linker**: Amino acid sequence between first and second RNA-binding domain of human protein kinase R
AVSPLLLTTTNSSEGLS
**TAT**
GRKKRRQRRRPQ
**FHV**
GRRRRNRTRRNRRRVRG

Supplementary Table 2: Details of G58T fusion proteins

siRNA Target	Source	Sequence (5′ to 3′)
**GAPDH (Human, Mouse and Rat)**	ThermoFisher Scientific, AM4631, 4457288,	Proprietary
**GAPDH (Mouse)**	Dharmacon, D-001930-02-05	Proprietary
**GAPDH (Human, Mouse)**	Custom-synthesized; Integrated DNA technology	Sense strand: CAGAAGACUGUGGAUGGCC
**HTT** (Mouse)**	Custom-synthesized, Integrated DNA technology	Sense strand: CCCUGGAAAAGCUGAUGACGG Sense Strand: CCGTGCAGATAAGAATGCTATAA Sense Strand: AGGGAATCAGAGGCAATTATTGG
**HTT siRNA #1** (Mouse)**	Dharmacon, L-040632-00-0005	Proprietary
**HTT siRNA #2* (Mouse)**	ThermoFisher Scientific MSS205084	Proprietary

** siRNA used *in vitro* and in the animal experiment. These siRNA were pooled together before adding to EVs. These siRNA specifically target mouse Htt gene in Q140 mice.

*smart pool mouse HTT siRNA used for i*n vitro* experiment in N2a cells.

Supplementary Table 3: Details of siRNAs used in this study.

Gene expression probes for RT-qPCR	Source	Catalogue
**GAPDH**	ThermoFisher Scientific	Hs02786624_g1GAPDH Hs02758991_g1 GAPDH Mm99999915_g1GAPDH
**HTT**	ThermoFisher Scientific	Mm01213820_m1
**HPRT**	ThermoFisher Scientific	Mm01324427_m1
**18S rRNA**	ThermoFisher Scientific	Mm03928990_g1 Hs99999901_s1
**ACTB**	ThermoFisher Scientific	Hs01060665_g1 Mm02619580_g1

Supplementary Table 4: Details of RT-qPCR assays used in this study.

Treatment Group	Composition of the formulation	Number of EVs /g body weight	Amount of siRNA
**Group I**	Saline	-	-
**Group II**	RVG EVs	(1.5– 2.0) ×10^{10}	-
**Group III**	EVG EVs +G58TF	(1.5– 2.0) ×10^{10}	-
**Group IV**	EVG EVs +G58TF+siRNA	(1.5– 2.0) ×10^{10}	10µg (0.5mg/kg dose)

Supplementary Table 5: Details of experimental groups for *in vivo* gene silencing experiments.

Primers used for inserting GAPDH gene into pET-28b (+) vector	
cDNA sequence of *GAPDH* gene<br>sequences in italics is a Flag tag.<br>Sequences underlined are a restriction endonuclease (NdeI and XhoI) sites used to insert the gene into the vector	CATATGGGGAAGGTGAAGGTCGGAGTCAACGGATTTGGTCGTATTGGGCGCCTGGTCACCAGGGCTGCTTTTAA<br>CTCTGGTAAAGTGGATATTGTTGCCATCAATGACCCCTTCATTGACCTCAACTACATGGTTTACATGTTCCAAT<br>ATGATTCCACCCATGGCAAATTCCATGGCACCGTCAAGGCTGAGAACGGGAAGCTTGTCATCAATGGAAATCCC<br>ATCACCATCTTCCAGGAGCGAGATCCCTCCAAAATCAAGTGGGGCGATGCTGGCGCTGAGTACGTCGTGGAGTC<br>CACTGGCGTCTTCACCACCATGGAGAAGGCTGGGGCTCATTTGCAGGGGGGAGCCAAAAGGGTCATCATCTCTG<br>CCCCCTCTGCTGATGCCCCCATGTTCGTCATGGGTGTGAACCATGAGAAGTATGACAACAGCCTCAAGATCATC<br>AGCAATGCCTCCTGCACCACCAACTGCTTAGCACCCCTGGCCAAGGTCATCCATGACAACTTTGGTATCGTGGA<br>AGGACTCATGACCACAGTCCATGCCATCACTGCCACCCAGAAGACTGTGGATGGCCCCTCCGGGAAACTGTGGC<br>GTGATGGCCGCGGGGCTCTCCAGAACATCATCCCTGCCTCTACTGGCGCTGCCAAGGCTGTGGGCAAGGTCATC<br>CCTGAGCTGAACGGGAAGCTCACTGGCATGGCCTTCCGTGTCCCCACTGCCAACGTGTCAGTGGTGGACCTGAC<br>CTGCCGTCTAGAAAAACCTGCCAAATATGATGACATCAAGAAGGTGGTGAAGCAGGCGTCGGAGGGCCCCCTCA<br>AGGGCATCCTGGGCTACACTGAGCACCAGGTGGTCTCCTCTGACTTCAACAGCGACACCCACTCCTCCACCTTT<br>GACGCTGGGGCTGGCATTGCCCTCAACGACCACTTTGTCAAGCTCATTTCCTGGTATGACAACGAATTTGGCTA<br>CAGCAACAGGGTGGTGGACCTCATGGCCCACATGGCCTCCAAGGAG*GATTACAAGGATGACGACGATAAGTAA*C<br>TCGAG
Gapdh(NdeI)fp	5' TTTATACATATGGGGAAGGTGAAGGTCGGAGTC 3'
Gapdh(XhoI)rp	5' TTTATTCTCGAGTTACTTATCGTCGTCATCCTTGTAATCCTCCTTGGAGGCCATGTGGGC<br>3'
Primers used for inserting Drosophila *GAPDH* 2 gene into pET-28b (+) vector	
cDNA sequence of *GAPDH* gene<br>(sequence in italics is a cMyc tag.)<br>Sequences underlined are a restriction endonuclease (NdeI and XhoI) sites used to insert the gene into the vector	CATATGTCGAAGATTGGTATCAATGGATTTGGTCGCATCGGCCGCTTGGTTCTCCGCGCCGCCATTGATAAGGG<br>CGCCAACGTTGTGGCCGTCAACGATCCCTTCATCGATGTGAACTACATGGTCTACCTGTTCAAGTTCGATTCGA<br>CCCACGGACGTTTTAAGGGCACCGTTGCCGCCGAGGGCGGTTTCCTGGTGGTCAACGGCCAGAAGATCACCGTC<br>TTCAGCGAACGCGACCCGGCCAACATCAACTGGGCCAGCGCTGGTGCCGAATACATCGTGGAGTCCACTGGCGT<br>GTTCACCACCATCGACAAGGCATCCACTCACTTGAAGGGCGGTGCCAAGAAGGTTATCATCTCGGCCCCATCCG<br>CCGATGCTCCCATGTTCGTTTGCGGCGTCAACTTGGATGCCTACAAGCCCGACATGAAGGTGGTCTCCAACGCA<br>TCGTGCACCACCAACTGCTTGGCTCCTCTGGCCAAGGTGATCAACGACAACTTCGAGATCGTCGAGGGTCTGAT<br>GACCACCGTTCATGCCACCACCGCTACCCAGAAGACCGTCGATGGACCTTCCGGCAAGTTGTGGCGTGATGGAC<br>GTGGCGCTGCCCAGAACATCATTCCAGCTTCCACTGGAGCTGCCAAGGCCGTGGGCAAGGTTATCCCCGCCCTC<br>AACGGTAAGCTCACCGGAATGGCATTCCGTGTGCCCACTCCCAACGTTTCCGTGGTCGATTTGACCGTGCGCTT<br>GGGCAAGGGTGCGTCCTATGATGAAATTAAGGCCAAGGTTCAGGAGGCCGCCAACGGACCCCTGAAGGGTATCC<br>TGGGATACACCGATGAGGAGGTCGTTTCTACCGATTTCCTCAGCGACACCCACTCGTCGGTGTTCGATGCCAAG<br>GCTGGCATTTCGCTAAACGACAAGTTCGTGAAGCTGATCTCTTGGTACGACAACGAGTTTGGCTACTCCAACCG<br>CGTCATCGACCTGATCAAGTACATGCAGAGCAAGGAT*GAACAAAAACTCATCTCAGAAGAGGATCTG*TAACTCG<br>AG
drosoGAPDH(NdeI)fp	5' TAATTACATATGTCGAAGATTGGTATC 3'
drosoGAPDH (XhoI)	5'TTTAAACTCGAGTTACAGATCCTCTTCTGAGATGAGTTTTTGTTCATCCTTGCTCTGCATGTACTTGATCAGGTC<br>3'

Supplementary Table 6 Details of GAPDH gene and primers used during cloning of GAPDH into pET28b vector

Primers used for constructing G58T, G150T, G58TF fusion genes	
G58(NdeI)fp	5' TTTATACATATGGGCACCGTCAAGGCTGAGAACG 3'
TRBP_G58rp	5' GCCACCGCCACCGACGTACTCAGCGCCAGCATC 3'
G58_TRBPf	5' TACGTCGGTGGCGGTGGCTCCCCTCAG 3'
TRBP_(XhoI)rp	5' TTATTACTCGAGTTAGCCATCCCGGGCATCCAGAGG 3'
G150(NdeI)fp	5' tttataCATATGgggaaggtgaaggtcggagtc 3'
TRBP_G150(150)rp	5' ACCGCCACCggcattgctgatgatcttgaggc 3'
G150_TRBPfp	5' GCCggtggcggtggctcccctcagcagtctgag 3'
G150(XhoI)rp	5' TTATTACTCGAGTTAGCCATCCCGGGCATCCAGAGGCAC 3'
G58TFfp(Ndei)fp	5' TTTATACATATGGGCACCGTCAAGGCTGAGAACG 3'
PKRL_FHV_G58TFrp	5' TAATAAAGGACTAACTGCGCCATCCCGGGCATCCAGAGGCAC 3'
G58T_PRKLFHVfp	5' GCCCGGGATGGC GCAGTTAGTCCTTTATTATTGACAACAACG 3'
FHV(XhoI)rp	5' TTATTACTCGAGTTATCCGCGCACACGTCTGCGATTCCTCCGAGTGCGATTACGCCG 3'

Supplementary Table 7 Sequence of primers used during cloning of G58T, G58TF and G150T constructs into pET28b vector

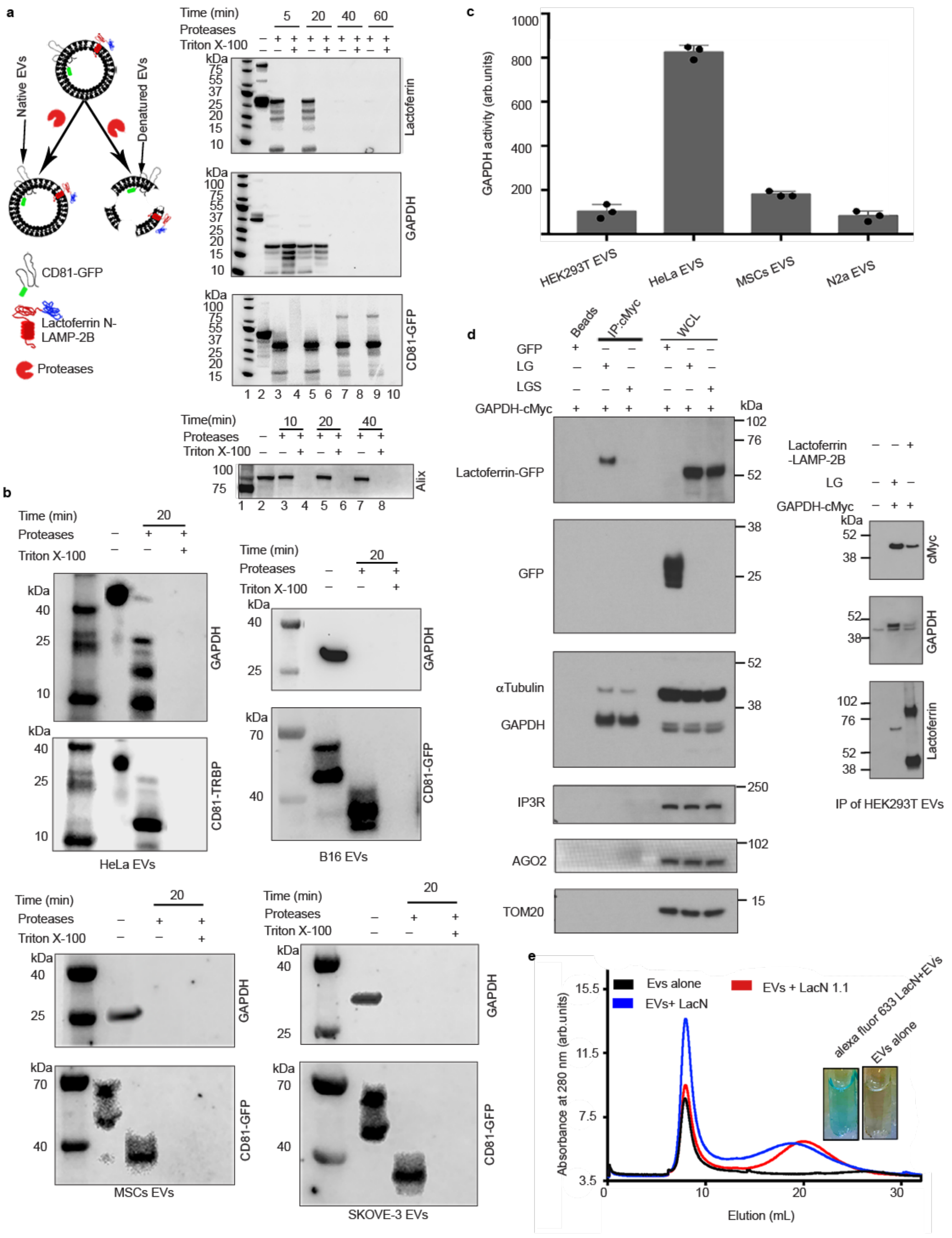

Supplementary Figure 1

GAPDH present on outer surface of EVs binds to cleaved lactoferrin.

a Protease digestion assay of EVs under native conditions and following detergent treatment (denatured conditions). Top left represents schematic drawing of protease digestion assay. CD81-GFP and Alix were used as positive control for protein present in the lumen of EVs. Western blotting images represent nature of lactoferrin, GAPDH, GFP and Alix proteins after

protease treatment. Digestion of lactoferrin and GAPDH bands under native conditions confirms their presence on EVs surface. Presence of intact Alix and GFP-containing band, which is partly truncated due to cleavage of an extracellular CD81 domain, under native conditions, confirms integrity of the membrane during the treatment. **b** Western blots of EVs from different cells after protease digestion assay showing digestion of EV GAPDH under native conditions. Digestion of GAPDH protein under native and denatured condition confirms presence on surface of EVs. **c** GAPDH enzyme kinetics of EVs from different cell sources. Equal numbers of EVs were used in the assay. The experiment were independently repeated 3 times. Results shown as mean±s.d. **d** Co-immunoprecipitation of lactoferrin and GAPDH protein expressed in HEK293T cells and EVs. Detection of lactoferrin-GFP band by the GFP antibody in lane 2 (top upper blot) confirms interaction of GAPDH protein with lactoferrin N domain. LG, lactoferrin-GFP protein; LGS, Lactoferrin-GFP with signal peptide of LAMP-2B. Attachment of a signal peptide to LG protein resulted in loss of lactoferrin interaction with the GAPDH for reasons that were not investigated; WCL, crude cell lysate used as positive control. Presence of α-tubulin bands shows interaction of GAPDH with α-tubulin. Insulin triphosphate receptor 3 (IPR3), Argonuate 2 (AGO2) and mitochondrial import receptor subunit TOM20 were used as negative controls. Western blot image on the right side shows co-immunoprecipitation of lactoferrin and GAPDH on surface of EVs. **e** Chromatogram of gel filtration chromatography showing absorbance of EVs at 280 nm after incubation with Lactoferrin N1.1 (LacN1.1) and Lactoferrin N (LacN) respectively. Inset represents EVs bound to Alexa Fluor-633 labelled lactoferrin N protein. Experiment **a** & **b** were repeated independently 2 times. Other experiments were repeated independently 3 times. Source data are provided as a Source Data file.

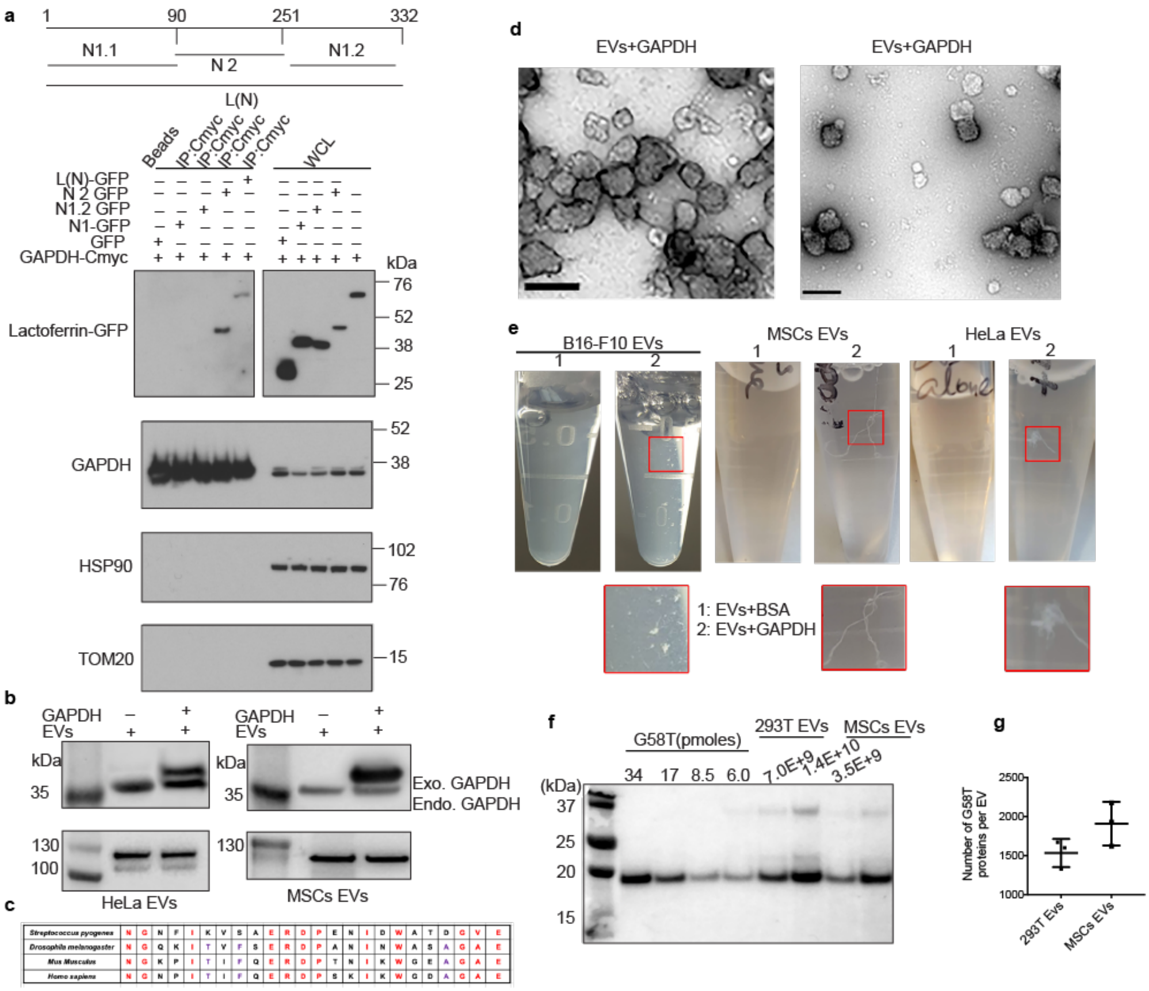

Supplementary Figure 2

Binding of exogenous GAPDH to EVs via G58 domain. a Co-immunoprecipitation assay showing interaction of different domains of lactoferrin N protein with GAPDH. Different domains of lactoferrin N are shown in schematic at the top of the figure. The lactoferrin N2 domain showed interaction with GAPDH. **b** Exogenous binding of GAPDH to EVs isolated from different cells. Higher molecular weight of exogenous GAPDH than endogenous GAPDH is due to presence of histidine tag (His_6) and flag tag. **c** Amino acid sequence of PS binding domain of GAPDH among different species. **d** Electron microscopic images of EVs treated with GAPDH protein, showing electron dense GAPDH protein tether around the EVs. (scale bar: 200nm). **e** Photographic images of the EVs after 2h incubation with either BSA or GAPDH protein. Addition of GAPDH lead to formation of conspicuous EV aggregates. **f** Western blot showing quantification of G58T protein on MSCs and HEK293T EVs (labelled as 293T EVs). 6.75×10^{11} EVs were incubated with 22 nmoles of G58T protein for 2h at 4˚C. Specific amounts of purified G58T protein were run on SDS-PAGE along with EV samples to determine the number of G58T proteins on EVs. **g** Scatter plot showing number of G58T proteins bound to each MSC and 293T EV as determined by quantification of immunoblots. Data shown in **g** as mean ± s.d (n=3). All experiments were independently repeated 3 times. Source data are provided as a Source Data file.

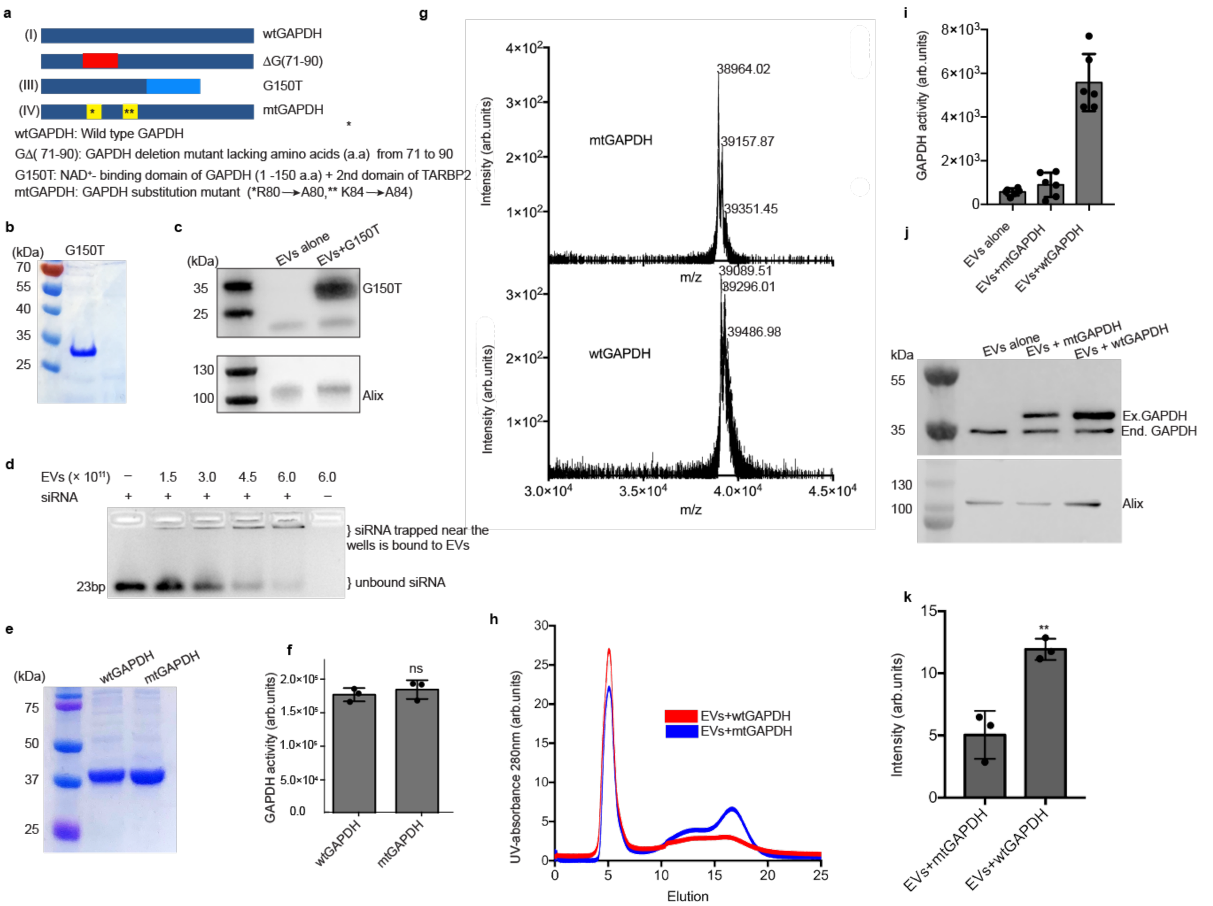

Supplementary Fig. 3: GAPDH mutation in the PS-binding domain affected EV binding of GAPDH without altering its glycolytic function: a Schematic shows different forms of GAPDH mutants. In G150T mutant, second domain of TARBP2 was attached for detecting protein via immunoblotting and carrying siRNA binding assay of the recombinant protein, GΔ (71-90); in this mutant PS-binding domain of GAPDH was deleted. The mutant protein could not be expressed due to some unknown reasons. **b & c** SDS PAGE and immunoblotting images representing the expression and binding of G150T protein to EVs respectively. **d** Agarose gel-shift assay of G150T bound EVs to assess their binding capacity. Gradual loss of siRNA band intensity at higher concentration of EVs reflects binding of G150T EVs to siRNA. **e** SDS PAGE of wtGAPDH and mtGAPDH after purifying them from BL21(DE3) cells. **f** GAPDH kinetic assay of wtGAPDH and mtGAPDH showing both proteins possess same enzymatic activity. Each dot is a representative of 3 independent experiments. Data shown as mean ± s.d (n=3). Statistical differences were determined by unpaired two-sided Student's t-test (ns; non-significant). **g** Matrix-assisted laser desorption/ionization-time of flight-mass spectrometry (MALDI-TOF-MS) of wtGAPDH and mtGAPDH showing their molecular mass, which is matching with their *in silico* molecular weight (wtGAPDH; 39211.50 Da. mtGAPDH; 39069.30 Da). **h** LC-chromatograph of EVs after incubation with either mtGAPDH or wtGAPDH. Higher absorbance of EVs incubated with wtGAPDH reflected more binding of wtGAPDH to EVs than mtGAPDH. **i** Bar chart showing GAPDH enzymatic assay of EVs after incubating them either with wtGAPDH or mtGAPDH. Reduction in GAPDH activity of EVs incubated with mtGAPDH reflected significant loss of

mtGAPDH binding to EVs. Each dot is a representative of 3 independent experiments. Data shown as mean ± s.d. Statistical differences were determined by unpaired two-sided Student's t-test,***p* = 0.0062 relative to EVs+ mtGAPDH) **j** Immunoblot of EVs showing level of wtGAPDH and mtGAPDH bound to the EVs. Equal number of EVs were loaded into the gel. Exo.GAPDH is exogenous GAPDH incubated with EVs. End. GAPDH represents endogenous GAPHD naturally associated with EVs. Higher molecular weight of Exo.GAPDH is due to Histidine tag and Flag tag attached to it. **k** Bar chart representing quantification of immunoblots of EVs incubated with either wtGAPDH or mtGAPDH. Data shown as mean ± s.d((n=3). Statistical differences were determined by unpaired two-sided Student's t-test, (***p*= 0.0048). All data shown were independently repeated 3 times. Source data are provided as a Source Data file

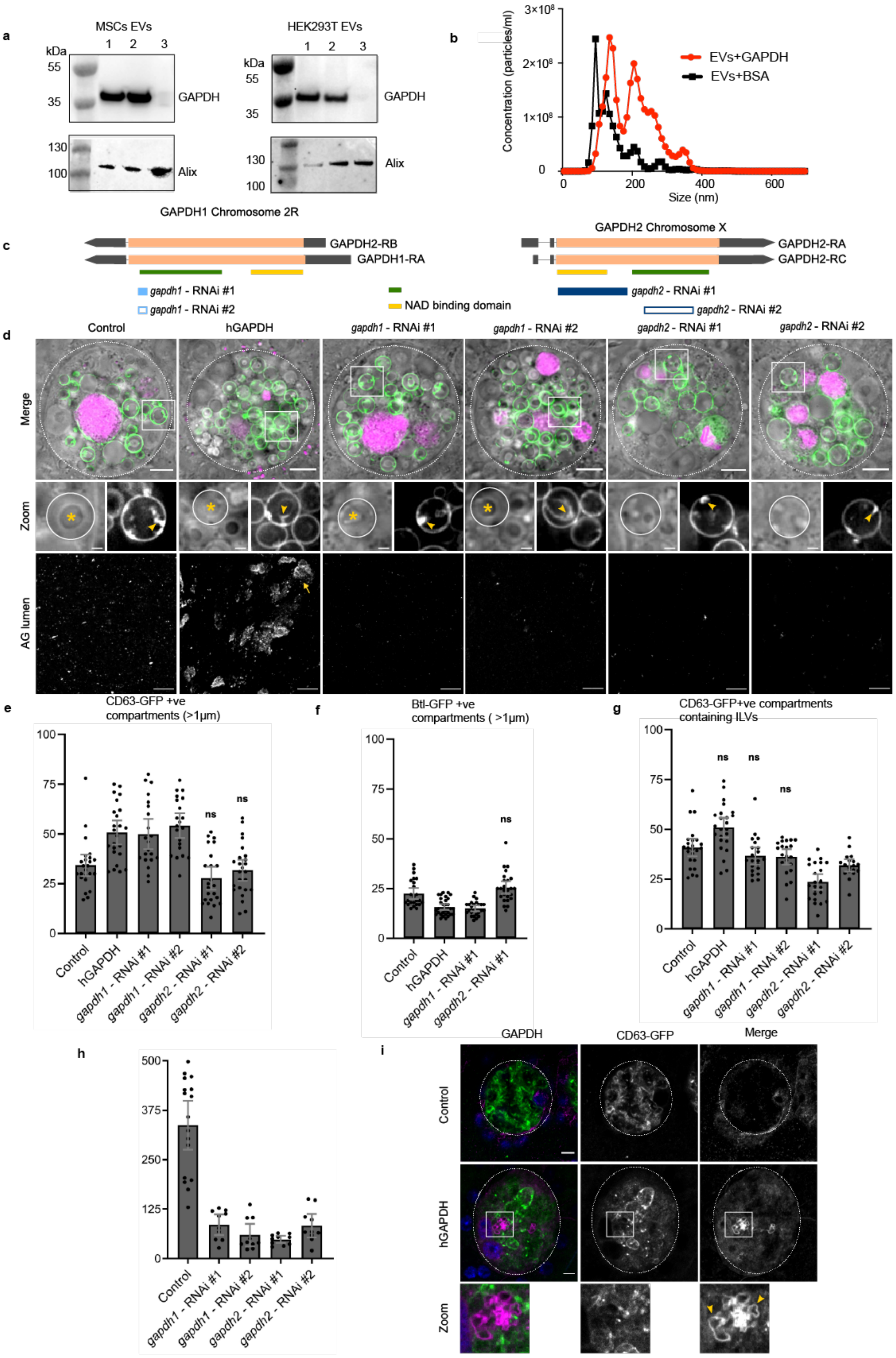
a
MSCs EVs
HEK293T EVs
kDa
55
35
130
100
1 2 3
GAPDH
Alix
b
Concentration (particles/ml)
3×10⁸
2×10⁸
1×10⁸
0
200
400
600
Size (nm)
EVs+GAPDH
EVs+BSA
c
GAPDH1 Chromosome 2R
GAPDH2 Chromosome X
GAPDH2-RB
GAPDH1-RA
GAPDH2-RA
GAPDH2-RC
gapdh1 - RNAi #1
gapdh1 - RNAi #2
NAD binding domain
gapdh2 - RNAi #1
gapdh2 - RNAi #2
d
Control
hGAPDH
gapdh1 - RNAi #1
gapdh1 - RNAi #2
gapdh2 - RNAi #1
gapdh2 - RNAi #2
Merge
Zoom
AG lumen
e
CD63-GFP +ve compartments (>1μm)
f
Btl-GFP +ve compartments (>1μm)
g
CD63-GFP+ve compartments containing ILVs
ns
h
500
375
250
125
i
GAPDH
CD63-GFP
Merge

Supplementary Fig. 4: Manipulating GAPDH levels in *Drosophila* secondary cells affects the biogenesis of CD63-GFP-labelled exosomes: a Western blot of MSC and 293T EVs after incubating with *Drosophila* GAPDH. 1; EVs+GAPDH+BSA, 2; EVs+GAPDH, 3; EVs alone **b** Nanosight tracking analysis of EVs after incubation with *Drosophila* GAPDH protein. **c** Schematic shows isoforms of *Drosophila* GAPDH1 and GAPDH2 and the targeted regions of each RNAi line used. Except for the *gapdh2*-RNAi #2, these RNAi lines do not have predicted off-targets. The two major protein domains, the highly conserved catalytic domain (green line) and the NAD binding domain (yellow line), are also shown. **d** Basal wide-field fluorescence and differential interference contrast ('Merge') views of living secondary cells (SCs) expressing GFP-tagged form of CD63 (CD63-GFP; green) with no other transgene (control); or also expressing the open reading frame of the human GAPDH protein (hGAPDH), two independent RNAi constructs targeting *Drosophila* GAPDH1 (*gapdh1* – RNAi #1 and #2), or two independent RNAi constructs targeting *Drosophila* GAPDH2 (*gapdh2* – RNAi #1 and #2) from eclosion onwards. SC outline approximated by dashed white circles, and acidic compartments are marked by LysoTracker Red (magenta). CD63-GFP-positive intraluminal vesicles (ILVs; green in 'Merge'; grey in 'Zoom') are apparent inside compartments, surrounding dense-core-granules (DCGs; asterisk in 'Zoom') and connecting DCGs to the limiting membrane of the compartment (yellow arrowheads, except in *GAPDH2* knockdown, where ILVs only surround peripheral small DCGs). DCG compartment outline is approximated by white circles. Panel also shows confocal transverse images of fixed accessory gland (AG) lumens from the same genotypes, containing CD63-GFP fluorescent puncta. **e** Bar chart shows average number of large (> 1 µm diameter) CD63-GFP-positive compartments per cell (hGAPDH, p=0.0016; *gapdh1* – RNAi #1, p=0.0077; *gapdh1* – RNAi #2, p=0.0002; *gapdh2* – RNAi #1, p>0.9999; *gapdh2* – RNAi #2, p>0.9999). **f** Bar chart, relating to Figure 2, shows average number of large (> 1 µm diameter) Btl-GFP-positive compartments per cell (hGAPDH, p=0.0004; *gapdh1* – RNAi #1, p=0.0002; *gapdh2* – RNAi #1, p=0.6198). **g** Bar chart shows the percentage of CD63-GFP-positive compartments per cell containing ILVs (*gapdh1* – RNAi #1, p=0.0022; *gapdh1* – RNAi #2, p<0.0001; *gapdh2* – RNAi #1, p<0.0001; *gapdh2* – RNAi #2, p=0.0017). **h** Bar chart shows number of CD63-GFP fluorescent puncta in the lumen of AGs for the different genotypes (hGAPDH, p=0.0978; *gapdh1* – RNAi #1, p>0.9999; *gapdh1* – RNAi #2, p>0.9999; *gapdh2* – RNAi #1, p<0.0001; *gapdh2* – RNAi #2, p=0.0470). Clustering of exosomes in the presence of hGAPDH prevented accurate quantification. **i** Basal confocal images of fixed SCs (n=4) isolated from males expressing Btl-GFP with no other transgene (control), or also expressing hGAPDH from eclosion onwards.

hGAPDH (magenta) and DAPI (blue) staining are shown. SC outline approximated by dashed white circles. GAPDH appears to associate with membranous structures inside late endosomal and lysosomal compartments when hGAPDH is overexpressed (yellow arrowheads in 'Zoom'). All data are from six-day-old male flies shifted to 29°C at eclosion to induce expression of transgenes. Genotypes in **d**, **e**, **g-i** are: *w; P[w⁺, UAS-CD63-GFP] P[w⁺, tub-GAL80ts]/+*; *dsx-GAL4/+* with no other transgene (control) (*n*=9 glands/*n*=24 cells) (*n*=17 AG lumens for ILV secretion), UAS-hGAPDH (*n*=7 glands/*n*=24 cells)(*n*=10 AG lumens), UAS-*gapdh1*-RNAi #1 (*n*=8 glands/*n*=21 cells)(*n*=10 AG lumens) and #2 (*n*=7 glands/*n*=21 cells)(*n*=10 AG lumens), UAS-*gapdh2*-RNAi #1 (*n*=7 glands/*n*=23 cells)(*n*=10 AG lumens) and #2 (*n*=7 glands/*n*=25 cells)(*n*=10 AG lumens).Genotypes in **f** are: *w; P[w⁺, tub-GAL80ts]/+*; *dsx-GAL4, P[w⁺, UAS-btl-GFP]/+* with no other transgene (control) (*n*=11 glands/*n*=24 cells), UAS-hGAPDH (*n*=10 glands/*n*=33 cells), UAS-*gapdh1*-RNAi #1 (*n*=10 glands/*n*=25 cells), and UAS-*gapdh2*-RNAi #1 (*n*=10 glands/*n*=25 cells). Scale bars in (b) (5 µm), in 'Zoom' (1 µm), and in 'AG lumen' (20 µm). *** p <0.001, ** p< 0.01, * p< 0.05, and ns, non-significant relative to control, Kruskal-Wallis followed by Dunn's multiple comparison test. Data shown in **e-h** as mean ± SEM (n=3). All experiments shown were independently repeated 3 times. Source data are provided as a Source Data file.

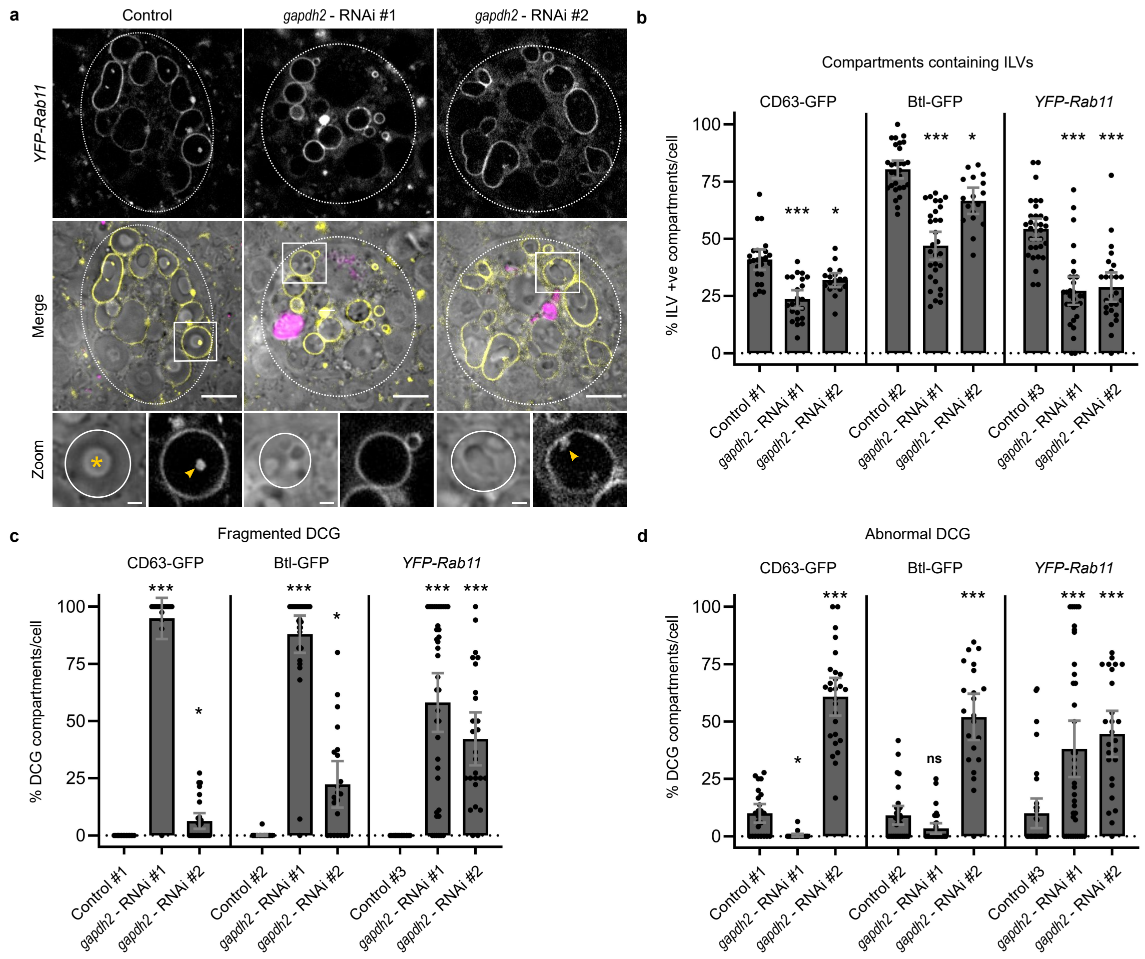

Supplementary Figure 5

***GAPDH2* knockdown affects exosome and DCG biogenesis in SCs, but not Rab11-compartment identity.**
a Basal wide-field fluorescence and differential interference contrast ('Merge') views of living secondary cells (SCs) expressing the *YFP-Rab11* gene trap (*YFP-Rab11*; yellow) with no other transgene (control), or also expressing either of two independent RNAi constructs targeting *Drosophila* GAPDH2 (*gapdh2* – RNAi #1 and #2) from eclosion onwards. SC outline approximated by dashed white circles, and acidic compartments are marked by LysoTracker Red (magenta). *YFP-Rab11*-positive intraluminal vesicles (ILVs; yellow in 'Merge'; grey in 'Zoom') are apparent inside compartments, but only near the compartment's limiting membrane in *GAPDH2* knockdown cells (yellow arrowheads). DCG compartment outline is approximated by white circles. **b** Bar chart showing the percentage of ILV-containing large (>1 µm diameter) compartments per cell marked with CD63-GFP (*gapdh2* – RNAi #1, p<0.0001; *gapdh2* – RNAi #2, p=0.0201), Btl-GFP (*gapdh2* – RNAi #1, p<0.0001; *gapdh2* – RNAi #2, p=0.0104) or YFP-Rab11 (*gapdh2* – RNAi #1, p<0.0001; *gapdh2* – RNAi #2,

p<0.0001). **c** Bar chart showing the percentage of DCG compartments per cell containing a fragmented DCG marked with CD63-GFP (*gapdh2* – RNAi #1, *p*<0.0001; *gapdh2* – RNAi #2, *p*=0.0466), Btl-GFP (*gapdh2* – RNAi #1, *p*<0.0001; *gapdh2* – RNAi #2, *p*=0.0093) or YFP-Rab11 (*gapdh2* – RNAi #1, *p*<0.0001; *gapdh2* – RNAi #2, *p*<0.0001). **d** Bar chart showing the percentage of DCG compartments per cell containing an abnormally shaped DCG marked with CD63-GFP (*gapdh2* – RNAi #1, *p*=0.0273; *gapdh2* – RNAi #2, *p*<0.0001), Btl-GFP (*gapdh2* – RNAi #1, *p*=0908; *gapdh2* – RNAi #2, *p*<0.0001) or YFP-Rab11 (*gapdh2* – RNAi #1, *p*=0.0003; *gapdh2* – RNAi #2, *p*<0.0001). All data are from six-day-old male flies shifted to 29°C at eclosion to induce expression of transgenes. Genotypes are: *w; P[w^{+}, UAS-CD63-GFP] P[w^{+}, tub-GAL80^{ts}]/+*; *dsx-GAL4/+* with no other transgene (control #1) (*n*=9 glands/*n*=24 cells), *w; P[w^{+}, tub-GAL80^{ts}]/+*; *dsx-GAL4, P[w^{+}, UAS-btl-GFP]/+* with no other transgenes (control #2) (*n*=11 glands/*n*=29 cells), *w; P[w^{+}, tub-GAL80ts]/+; dsx-GAL4, TI{TI}Rab11EYFP/+* with no other transgene (control #3) (*n*=14 glands/*n*=33 cells), or the same genotypes with UAS-*gapdh2*-RNAi #1 (CD63-GFP: *n*=7 glands/*n*=23 cells ;Btl-GFP: *n*=10 glands/*n*=34 cells; YFP-Rab11: *n*=12 glands/*n*=38 cells)and #2 (CD63-GFP: *n*=7 glands/*n*=27 cells ;Btl-GFP: *n*=7 glands/*n*=22 cells; YFP-Rab11: *n*=7 glands/*n*=26 cells). Scale bars in **(a)** (5 µm) and in 'Zoom' (1 µm). ****p*<0.001, ***p*<0.01, **p*<0.05, and ns, non-significant relative to control, Kruskal-Wallis followed by Dunn's multiple comparison test. Data shown in **b-d** as mean ± SEM (n=3). All experiments shown were independently repeated 3 times. Source data are provided as a Source Data file.

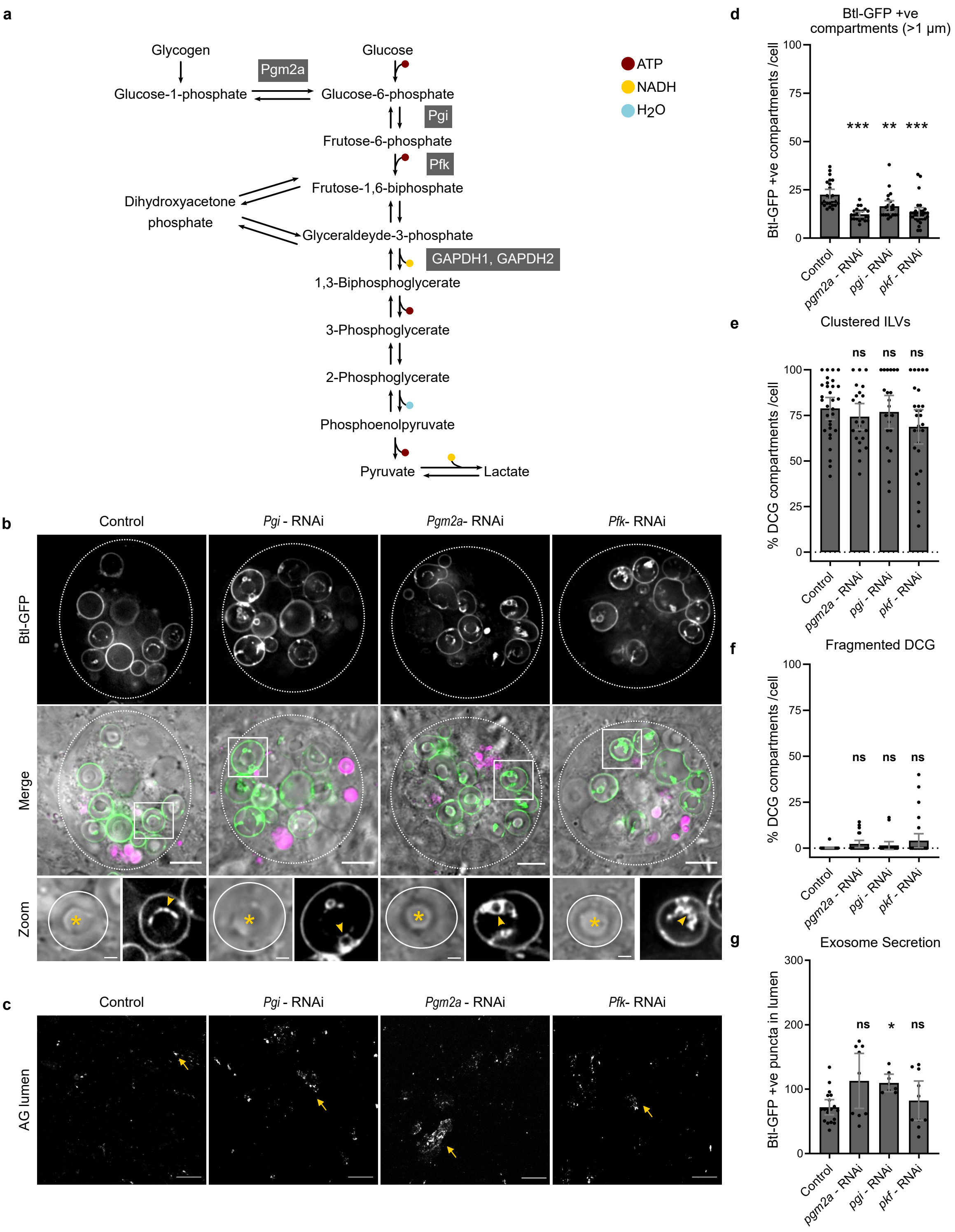

Supplementary Figure 6

The effects of knocking down other glycolytic enzymes on exosome biogenesis and clustering in *Drosophila* secondary cells.

a Schematic shows a simplified glycolytic pathway, in which the three glycolytic enzymes that were analysed in this study, GAPDH1 and GAPDH2, Pgi and Pgm2a, are highlighted. **b** Basal wide-field fluorescence and differential interference contrast ('Merge') views of living

secondary cells (SCs) expressing a GFP-tagged form of Breathless (Btl-GFP; green) with no other transgene (control); or also expressing an RNAi construct targeting *Drosophila* Phospho-glucose isomerase (*Pgi* - RNAi), Phosphoglucomutase 2a (*Pgm2a* - RNAi) or Phosphofructokinase (*Pfk* - RNAi) from eclosion onwards. SC outline approximated by dashed white circles, and acidic compartments are marked by LysoTracker Red (magenta). Btl-GFP-positive intraluminal vesicles (ILVs; green in 'Merge'; grey in 'Zoom') are apparent inside compartments (yellow arrowheads), surrounding dense-core-granules (DCGs; asterisk in 'Zoom'). DCG compartment outline is approximated by white circles. **c** Confocal transverse images of fixed accessory gland (AG) lumens from the same genotypes, containing Btl-GFP fluorescent puncta. **d** Bar chart shows average number of large (>1 μm diameter) Btl-GFP-positive compartments per cell (*Pgi* - RNAi, p=0.0098; *Pgm2a* - RNAi, p<0.0001; *Pfk* - RNAi, p<0.0001). **e** Bar chart shows percentage of DCG compartments per cell containing clustered Btl-GFP-positive ILVs that are in contact with DCGs (*Pgi* - RNAi, p>0.9999; *Pgm2a* - RNAi, p=0.9048; *Pfk* - RNAi, p=0.3402). **f** Bar chart shows the percentage of DCG compartments per cell containing a fragmented DCG (*Pgi* - RNAi, p=0.0015; *Pgm2a* - RNAi, p<0.0001; *Pfk* - RNAi, p<0.0001). **g** Bar chart shows Btl-GFP fluorescent puncta in the lumen of AGs from the same genotypes. All data are from six-day-old male flies shifted to 29°C at eclosion to induce expression of transgenes. Genotypes are: *w; P[w$^+$, tub-GAL80^{ts}]/+*; *dsx-GAL4, P[w$^+$, UAS-btl-GFP]/+* with no other transgene (control) (n=11 glands/n=24 cells) (n=10 AG lumens for ILV secretion), or with UAS-*Pgi*-RNAi (n=7 glands/n=23 cells) (n=10 AG lumens), UAS-*Pgm2a*-RNAi (n=7 glands/n=22 cells) (n=10 AG lumens), or UAS-*pfk*-RNAi (n=10 glands/n=30 cells) (n=10 AG lumens). Scale bars in **(b)** (5 μm), in 'Zoom' (1 μm), and in 'AG lumen' (20 μm). ***p<0.001, **p<0.01 and *p<0.05 relative to control, Kruskal-Wallis followed by Dunn's multiple comparison test. Data shown in **d-g** as mean ± SEM(n=3). All experiments shown were independently repeated 3 times. Source data are provided as a Source Data file.

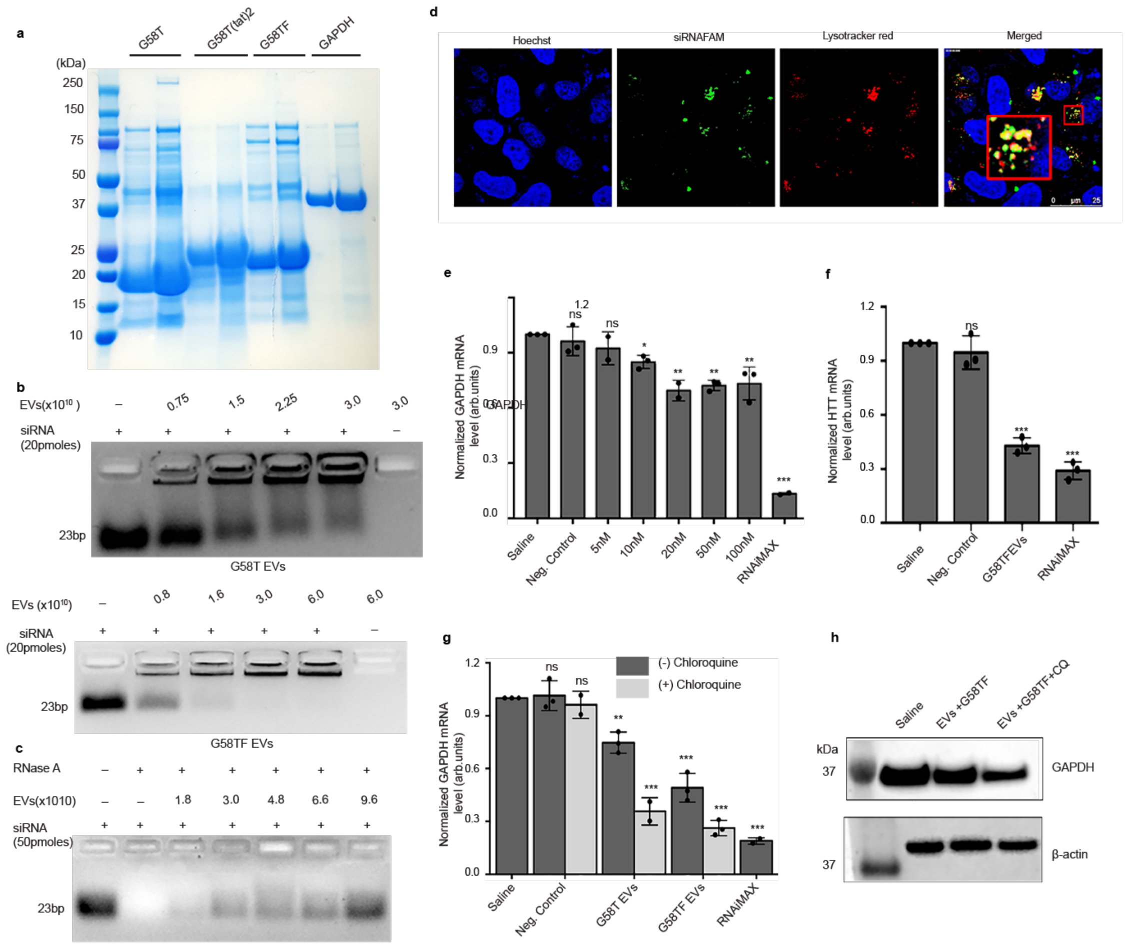

Supplementary Figure 7

G58T-mediated loading of siRNA on EVs.

a SDS-PAGE of the G58T-based proteins purified by Ni-NTA chromatography. Gel is stained with Coomassie brilliant blue dye. **b** Gel shift assay of G58T EVs (upper) and G58TF EVs (lower), reflecting binding of siRNA to EVs. Bands trapped near the wells represent bound siRNA. siRNA alone was used as negative control. **c** RNase A protection assay of G58TF EVs bound to 50 pmol of siRNA. Bands in the agarose gel represent siRNA isolated from G58TF EVs after treatment with RNase A (0.2 mg/ml). **d** Representative confocal microscopy images of N2a cells after 4 h of treatment with G58TF EVs carrying FAM-labelled siRNA (green). Cells were treated with lysotracker red dye (red dots) to label late-endosomes. Inset in the merged figure represents magnified image of N2a cells showing colocalization of siRNA with lysotracker dye (yellow spots). Images were captured using 60× objective lens of Olympus confocal microscope FV1000. **e** GAPDH gene silencing by G58T EVs at mRNA level in N2a cells. *p = 0.0371, **p= 0.0005, ***p=0.0001. **f** Bar graph represents silencing of HTT gene at mRNA level in N2a cells after 48h of treatment with 50 nM HTT siRNA bound to G58TF EVs. ***p= 0.0001 when compared to control. **g** Bar graph showing effect of chloroquine on silencing of GAPDH at mRNA level by G58T and G58TF EVs in N2a cells. **p=0.006, ***p =0.0001 when compared to control **h** Western blot showing GAPDH silencing in HeLa cells by G58TF EVs in presence and absence of

chloroquine (30 µM). Data shown in **e-g** as mean ± s.d (n=3). Statistical differences in **e-g** were determined by one way ANOVA, using Dunnett's multiple comparisons test. All the experiments shown were independently repeated 3 times. Source data are provided as a Source Data file.

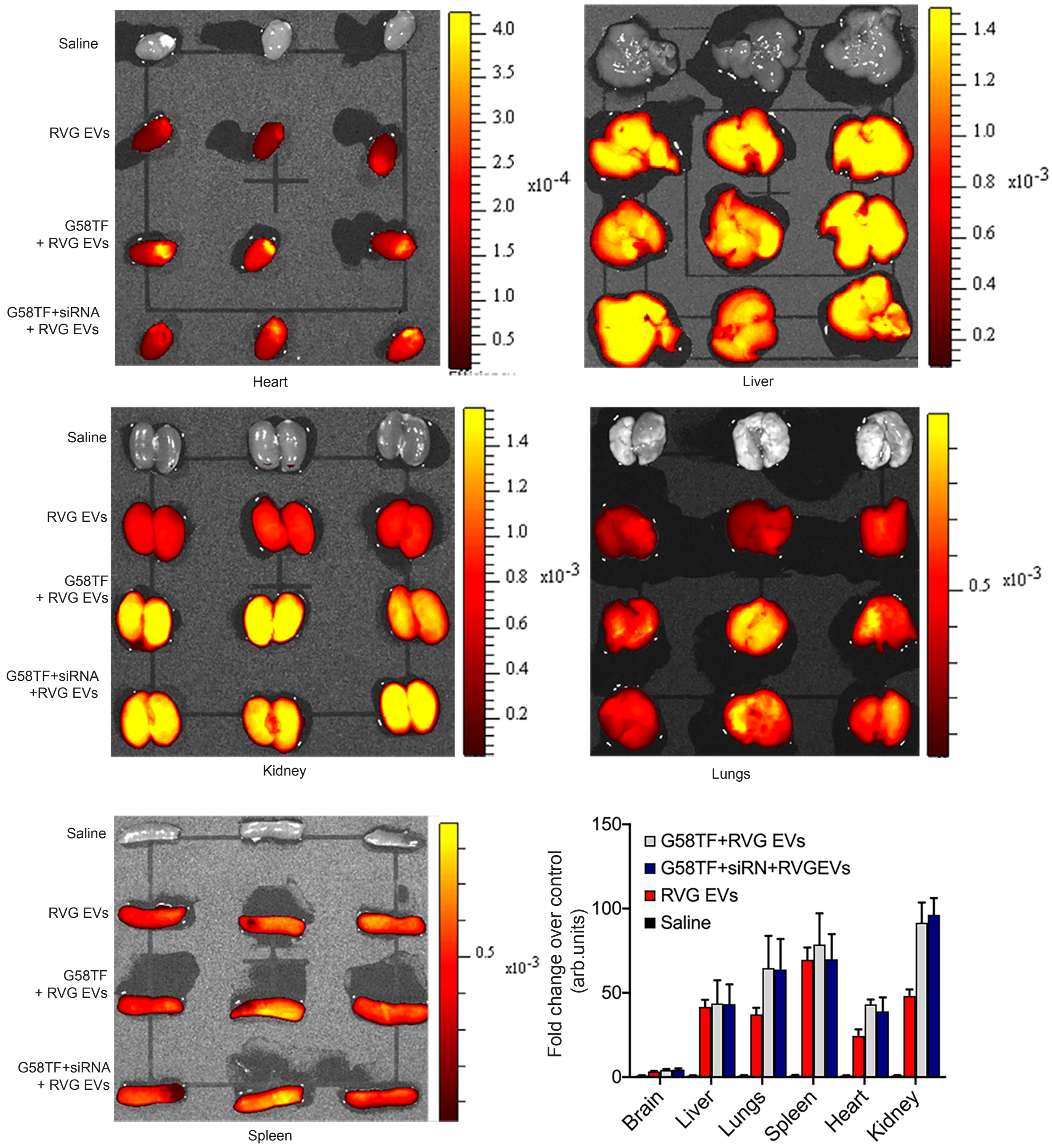

Supplementary Figure 8

Biodistribution of systemically administered modified EVs.

In vivo animal imaging of C57BL/6 animals after 4 h of administration of G58TF EVs labelled

with Cy5.5 dye. Images represent fluorescence of Cy5.5 dye in various organs of the animals. Graph shows quantification of the signals in different organs of the animals. Results are shown as mean±s.d (n=3 mice).

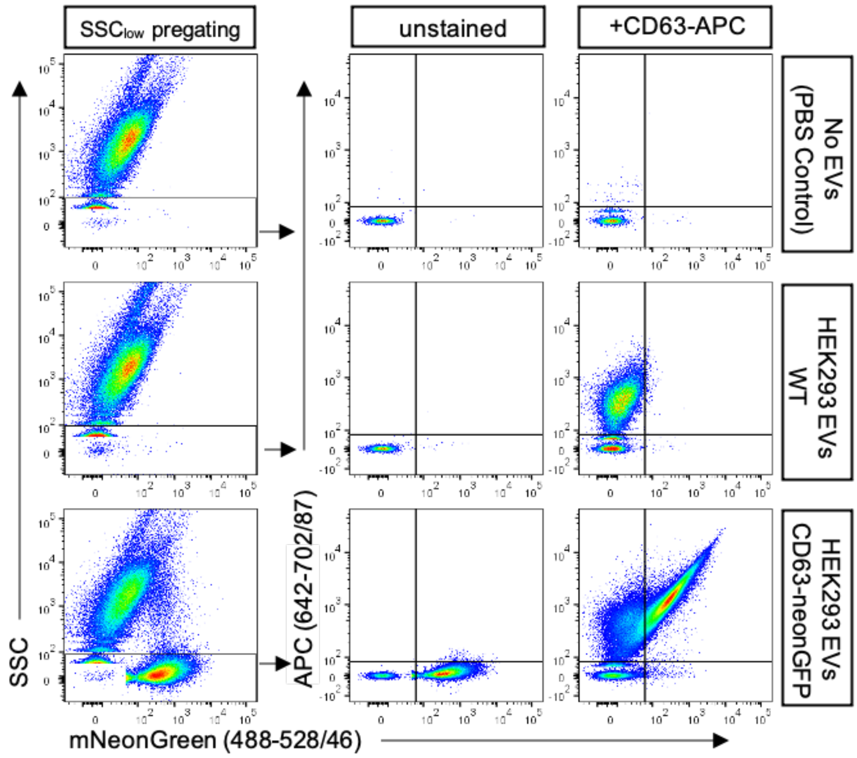

Supplementary figure 9.

Gating strategy applied for imaging flow cytometry analyses. All samples were pregated on SSC (low) with a cut-off chosen based on scattering properties of 293F-derived CD63-mNeonGreen tagged EVs which were described as biological reference before (bottom left graph; Görgens et al, JEV 2019). Examples include non-EV containing buffer control (PBS+antibody; top row), WT EVs unstained and stained with CD63-APC antibodies (middle row), and CD63-mNeonGreen tagged EVs unstained and stained with CD63-APC antibodies. Gates for fluorescence positivity were set based on single stained controls, respectively. The same gating strategy was applied to all other samples analyzed.
